# Development of an Electronic Frailty Index for Predicting Mortality and Complications Analysis in Pulmonary Hypertension Using Random Survival Forest Model

**DOI:** 10.3389/fcvm.2022.735906

**Published:** 2022-07-08

**Authors:** Jiandong Zhou, Oscar Hou In Chou, Ka Hei Gabriel Wong, Sharen Lee, Keith Sai Kit Leung, Tong Liu, Bernard Man Yung Cheung, Ian Chi Kei Wong, Gary Tse, Qingpeng Zhang

**Affiliations:** ^1^Nuffield Department of Medicine, University of Oxford, Oxford, United Kingdom; ^2^Frailty Assessment Unit, Cardiovascular Analytics Group, Hong Kong, Hong Kong SAR, China; ^3^Division of Clincal Pharmacology, Department of Medicine, School of Clinical Medicine, Li Ka Shing Faculty of Medicine, University of Hong Kong, Hong Kong, Hong Kong SAR, China; ^4^Aston Medical School, Aston University, Birmingham, United Kingdom; ^5^Tianjin Key Laboratory of Ionic-Molecular Function of Cardiovascular Disease, Department of Cardiology, Tianjin Institute of Cardiology, Second Hospital of Tianjin Medical University, Tianjin, China; ^6^Department of Pharmacology and Pharmacy, The University of Hong Kong, Hong Kong, Hong Kong SAR, China; ^7^Medicines Optimisation Research and Education, UCL School of Pharmacy, London, United Kingdom; ^8^Kent and Medway Medical School, Canterbury, United Kingdom; ^9^School of Data Science, City University of Hong Kong, Hong Kong, Hong Kong SAR, China

**Keywords:** pulmonary hypertension, electronic frailty index, random survival forest (RSF), diabetes mellitus, cardiovascular disease, renal complications

## Abstract

**Background:**

The long-term prognosis of the cardio-metabolic and renal complications, in addition to mortality in patients with newly diagnosed pulmonary hypertension, are unclear. This study aims to develop a scalable predictive model in the form of an electronic frailty index (eFI) to predict different adverse outcomes.

**Methods:**

This was a population-based cohort study of patients diagnosed with pulmonary hypertension between January 1st, 2000 and December 31st, 2017, in Hong Kong public hospitals. The primary outcomes were mortality, cardiovascular complications, renal diseases, and diabetes mellitus. The univariable and multivariable Cox regression analyses were applied to identify the significant risk factors, which were fed into the non-parametric random survival forest (RSF) model to develop an eFI.

**Results:**

A total of 2,560 patients with a mean age of 63.4 years old (interquartile range: 38.0–79.0) were included. Over a follow-up, 1,347 died and 1,878, 437, and 684 patients developed cardiovascular complications, diabetes mellitus, and renal disease, respectively. The RSF-model-identified age, average readmission, anti-hypertensive drugs, cumulative length of stay, and total bilirubin were among the most important risk factors for predicting mortality. Pair-wise interactions of factors including diagnosis age, average readmission interval, and cumulative hospital stay were also crucial for the mortality prediction. Patients who developed all-cause mortality had higher values of the eFI compared to those who survived (*P* < 0.0001). An eFI ≥ 9.5 was associated with increased risks of mortality [hazard ratio (HR): 1.90; 95% confidence interval [CI]: 1.70–2.12; *P* < 0.0001]. The cumulative hazards were higher among patients who were 65 years old or above with eFI ≥ 9.5. Using the same cut-off point, the eFI predicted a long-term mortality over 10 years (HR: 1.71; 95% CI: 1.53–1.90; *P* < 0.0001). Compared to the multivariable Cox regression, the precision, recall, area under the curve (AUC), and C-index were significantly higher for RSF in the prediction of outcomes.

**Conclusion:**

The RSF models identified the novel risk factors and interactions for the development of complications and mortality. The eFI constructed by RSF accurately predicts the complications and mortality of patients with pulmonary hypertension, especially among the elderly.

## Introduction

Pulmonary hypertension (PHTN) was defined in the First World Symposium on Pulmonary Hypertension as having a mean pulmonary arterial pressure >25 mmHg at resting by right heart catheterization (1, 2). PHTN was traditionally classified as either primary or secondary PHTN (3). The epidemiology and prognosis of the PHTN vary with different causes, but generally, PHTN can progress to a severe stage and ultimately cause death if left untreated (4). Therefore, it is essential to evaluate the prognosis of patients with PHTN as early as possible. Nevertheless, the prognostic risk factors to predict the risks of complication development and mortality are unclear.

Currently, the PHTN mortality is predicted using the dynamic risk stratification strategy suggested in the European Society of Cardiology/European Respiratory Society pulmonary hypertension guidelines. The strategy utilizes the clinical features and laboratory results to predict the mortalities and stratify the risk of death according to the one-year mortality expectations (5, 6). However, the model requires extensive clinical investigations, such as echocardiography and cardiopulmonary exercise testing (7). Calculating the risks of death by simply using the demographics and the laboratory testing results allows the determination of the treatment objectives to be more readily accessible.

PHTN can result in serious complications involving the cardiovascular, renal, and metabolic systems (8). Conversely, patients with pre-existing comorbidities have a poorer prognosis (9, 10). For example, acute right heart failure is one of the most important causes of mortality among patients with PHTN (11). PHTN can also co-exist with chronic renal disease owing to altered fibroblast growth factor-23 signaling (12, 13). The presence of diabetes mellitus can induce pulmonary endothelial dysfunction, and patients with PHTN may also develop diabetes mellitus and metabolic syndrome as complications due to the chronic pro-inflammatory states (14, 15). The concurrence of the above conditions increases the complexity of the PHTN clinical profile.

Frailty is a geriatric syndrome that results in age-associated functional limitations across multiple systems. Older people with frailty are prone to poorer health outcomes such as falls and disability. This contributes to frequent hospitalization and premature death (16–19). The development of an electronic frailty index (eFI) through the random survival forest (RSF) model allows the analysis of the survival data using electronic health data (20). RSF can approximate complex survival functions while maintaining low prediction error (21). This study aims to construct a scalable eFI with improved predictability for complications and short-term mortality among patients with PHTN through the application of the RSF model.

## Methods

### Study Design and Population

The retrospective population-based cohort was designed to investigate long-term clinical prognostic risk factors that predict the survival of patients with newly diagnosed PHTN. This cohort included patients diagnosed with PHTN between January 1st, 2000 and December 31st, 2017 at centers managed by the Hong Kong Hospital Authority. The patients were identified from the Clinical Data Analysis and Reporting System (CDARS), a territory-wide database that centralizes patient information from individual local hospitals to establish comprehensive medical data. This system has previously been used by local teams to conduct population-based epidemiological studies (22, 23), including the development of eFIs (24, 25).

Demographics, comorbidities, hospitalization characteristics, drug prescriptions, and laboratory examinations at the baseline were extracted. Drug prescriptions following the diagnosis of PHTN were determined. The calculated mean daily prescribed drug dosage of medications was noted. The mean daily dose of each drug class is derived from multiplying the daily dose frequency by the drug dose then averaged by the cumulative duration. Details regarding the International Classification of Diseases, Nineth Edition (ICD-9) codes for identifying the comorbidities and PHTN drugs are provided (Supplementary Tables 1, 2).

### Statistical Analysis

The study outcomes were the development of cardiovascular and renal complications, diabetes mellitus, and mortality after diagnosing PHTN. The mortality data were obtained from the Hong Kong Death Registry, a population-based official government registry with the registered death records of all Hong Kong citizens. Continuous variables were represented as median (95% confidence interval [CI] or interquartile range [IQR]), and categorical variables were presented count (%). Continuous variables were compared using the Mann-Whitney U test. The χ2 test with Yates' correction was utilized for 2 × 2 contingency data. The Pearson's χ2 test was applied for variables with over two categories of contingency data.

The univariable Cox model was used to uncover the significant prognostic risk factors associated with the outcomes *via* adjustments based on baseline characteristics. Significantly predictive factors are used as input of the multivariable Cox regression and the RSF analysis model for the complication prediction. Hazard ratios (HRs) with corresponding 95% CIs and *P*-values were reported. All significance tests were two tailed and considered significant if *P* < 0.05. Data analyses were performed using the RStudio software (Version: 1.1.456) and Python (Version: 3.6).

### Development of a Tree-Based Mortality Prediction Model

The eFI was developed to use the primary electronic health record to predict the frailty status of the patients, based on the principle that it reflects the health deficit accumulation (26, 27). Our team has previously developed an eFI for heart failure (24). A survival analysis was utilized to estimate the probability of mortality after diagnosing PHTN. It identifies the most influential prognostic risk factors that efficiently predict mortality outcomes. The RSF model was used to conduct a supervised survival learning analysis with the electronic health data. We used 20% of the data as a test set for a model performance evaluation and comparisons while using the remaining 80% for a model training. The parameters that gave the highest value of the Concordance Index (C-index) on the test set were chosen as the final model. The RSF was employed to learn the distribution of survival times based on the observed preoperative symptom data.

### Variable Importance Measure

The variable importance value of each factor was calculated to investigate the predictive strength. We leave out about 40% of instances whenever a bootstrap sample is down with replacement from the training data set. These left-out instances are referred to as out-of-bag (OOB) ones and the instances in the bootstrap sample as in bag ones. To calculate the variable importance value, we dropped each OOB instance down its in-bag competing risk tree and assigned a child node randomly whenever a split for the variable is encountered. The event-specific cumulative probability function from each such tree is calculated and averaged. The importance value is the prediction error for the original ensemble event-specific cumulative probability function (obtained when each OOB instance is dropped down its in-bag competing risks tree) subtracted from the prediction error for the new ensemble obtained using randomizing assignments of the variable (28, 29). The prediction errors are computed using squared loss. A higher importance value indicates higher predictive strength of the variable, whereas zero or negative values indicate non-predictive variables.

### Minimal Depth Approach

Minimal depth (30) ranks the variables through the inspection of the forest construction process under the assumption that variables with high impact on the prediction are those that most frequently split nodes nearest to the root node, where they partition the largest samples. Within each split tree, minimal depth approaches numbers of node levels according to their relative distance to the root of the tree (with the root node at 0). In such a way, the minimal depth approach can identify important variables by averaging the depth of the first split for each variable over all trees within the final forest.

The minimal depth approach was used to capture the variable interactions. We calculate the importance measures of pairwise interactions among variables since the minimal depth measure is defined by averaging the tree depth of the variable of interest relative to the root node. To compute the interaction strength for prediction, this calculation is modified to measure the minimal depth of a variable xi with respect to the maximal subtree for variable xj.

In general, to select the most influential variables with a variable importance approach, we examine the calculated variable importance values. The minimal depth approach is a non-event-specific criterion, whereas the variable importance approach can be both event specific and non-event specific. In the following analysis, we used the mortality-specific and time-to-event-specific variable importance.

### Performance Evaluation

The 5-fold cross validation approach is adopted to evaluate the prediction performance of the RSF model. The metrics of precision and recall are as follows:


$$\begin{array}{l} Precision=\frac{TP}{TP+FP},\;Recall=\frac{Tp}{Tp+FN}, \end{array}$$

and the area under the receiver operating characteristic curve (AUC) and the C index are used for performance evaluation. *TN*, *TP*, *FP*, and *FN* represent true positive, true negative, false positive, and false negative rates in the confusion matrix, respectively. Conventional non-parametric survival models primarily select the best analysis model that maximizes the C index (31). C index quantifies the degree to which the predicted outcomes in the pairwise orderings are consistent with the observed outcomes and can be regarded as a generalization of the AUC. That is, any survival analysis model that properly estimates the ordered but proportional event times can score high in terms of C index. C index can be used as a global assessment of the model's discrimination power: the ability to correctly provide a reliable ranking of the survival times based on the individual risk scores. In this study, we compare the RSF model with the traditional Cox analysis to predict the mortality and complications after diagnosis of PHTN.

## Results

### Basic Characteristics

**Figure 1:**
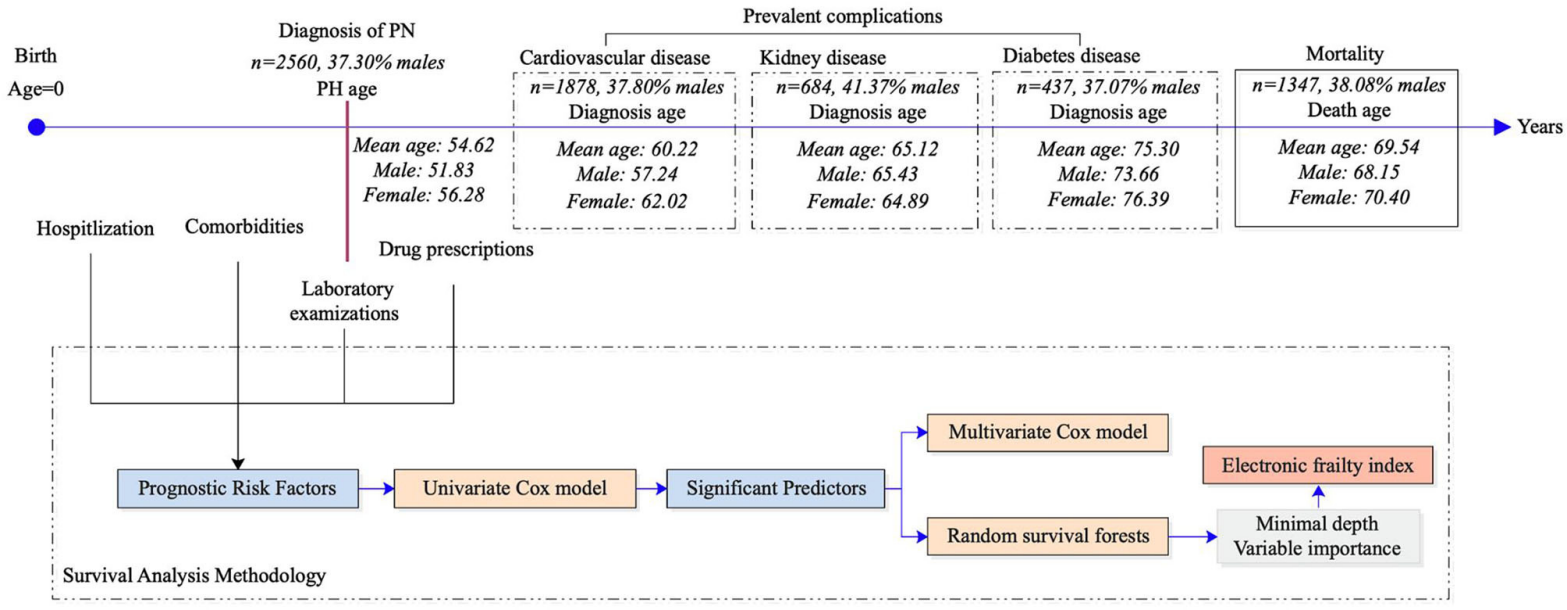
Illustration of the study procedures. PN, Pulmonary hypertension.

The study cohort included 2,560 patients (37% men) with an age of 63.4 (IQR: 38.0–79.0) (Figure 1). The baseline characteristics of the PHTN patients are detailed in Tables 1, 2. Over the follow-up, 38% of patients died; those who died were older (median: 75.2, IQR: 60.0–83.0) than those who survived (median: 44.6, IQR: 1.0–65.0).

**Table 1:** The characteristics of patients with pulmonary hypertension stratified by mortality outcomes.

**Characteristics**	**All patients (*n* = 2,560) Median (IQR) or count (%)**	**Mortality (*n* = 1,347)** ** Median (IQR) or count (%)**	**No mortality (*n* = 1,213) Median (IQR) or count (%)**	***P*-value**
**Demographics**				
Male sex	955 (37.30%)	513 (38.08%)	442 (36.43%)	<0.0001^***^
Age at diagnosis	63.4 (38.0–79.0)	75.2 (60.0–83.0)	44.6 (1.0–65.0)	<0.0001^***^
**Hospitalization before PHTN**				
Total number of hospital admissions	13.0 (7.0–23.0)	16.0 (8.0–26.0)	11.0 (5.0–20.0)	<0.0001^***^
Number of emergency readmissions	3.0 (1.0–6.0)	4.0 (2.0–8.0)	2.0 (1.0–4.0)	0.0115^*^
Mean readmission interval (days)	223.7 (105.0–435.0)	221.3 (115.0–387.0)	225.4 (95.0–509.0)	0.1725
Cumulative length-of-stay	80.0 (34.0–162.0)	110.0 (58.0–191.0)	48.0 (21.0–118.0)	<0.0001^***^
**Comorbidity before PHTN**				
Respiratory disease	2,537 (99.10%)	1,330 (98.73%)	1,207 (99.50%)	0.2782
Hypertension	2,511 (98.08%)	1,313 (97.47%)	1,198 (98.76%)	0.0617
Cardiovascular disease	1,916 (74.84%)	1,150 (85.37%)	766(63.14%)	<0.0001^***^
Gastrointestinal disease	1,014 (39.60%)	601 (44.61%)	413 (34.04%)	0.0016^**^
Kidney disease	768 (30.00%)	534 (39.64%)	234 (19.29%)	<0.0001^***^
Endocrine disease	88 (3.43%)	44 (3.26%)	44 (3.62%)	0.0916
Diabetes mellitus	337 (13.16%)	218 (16.18%)	119 (9.81%)	<0.0001^***^
Obesity	71 (2.77%)	29 (2.15%)	42 (3.46%)	<0.0001^***^
**Drug prescriptions after PHTN**				
**Alpha blockers**	***n*** **= 224**	***n*** **= 159**	***n*** **= 65**	
Daily dosage, mg/day	1.7 (1.0–3.0)	1.7 (0.9, 3.0)	1.9 (1.1, 3.5)	*p* = 0.2443^***^
Cumulative dosage, mg	377.5 (84.0, 1165.0)	258.5 (49.5, 924.5)	543.5 (177.0–2319.5)	0.0016^**^
Cumulative duration, days	260.5 (62.0–753.5)	237.0 (40.0–696.0)	396.0 (112.0–1141.0)	0.0648
**Anti-arrhythmias drugs**	***n*** **= 238**	***n*** **= 179**	***n*** **= 59**	
Daily dosage, mg/day	225.0 (46.5–636.5)	225.0 (48.0–661.5)	117.0 (20.0–584.5)	0.2261
Cumulative dosage, mg	227.86 (36.0–1524.11)	255.0 (37.4–1660.55)	182.81 (24.0–1023.75)	0.1277
Cumulative duration, days	4.0 (2.0–17.5)	4.0 (2.0–12.5)	5.0 (2.0–35.0)	0.582
**Beta blockers**	***n*** **= 670**	***n*** **= 402**	***n*** **= 268**	
Daily dosage, mg/day	15.76 (6.25–31.41)	13.26 (6.23–26.36)	18.99 (6.26–41.97)	0.0611
Cumulative dosage, mg	784.0 (125.0–4948.44)	575.0 (76.5–3966.0)	1599.5 (187.0–8473.44)	0.0001^***^
Cumulative duration, days	178.0 (37.0–499.0)	133.0 (26.0–397.0)	280.0 (54.0–867.0)	<0.0001^***^
**Cardiac glycosides**	***n*** **= 491**	***n*** **= 362**	***n*** **= 129**	
Daily dosage, mg/day	62.5 (42.85–144.34)	63.14 (48.28–148.2)	57.34 (25.79–125.0)	0.0111^*^
Cumulative dosage, mg	4437.5 (371.0–23625.0)	5181.25 (562.5–19250.0)	2662.5 (155.0–45562.5)	0.7103
Cumulative duration, days	172.0 (24.0–551.5)	154.0 (21.0–473.0)	252.0 (30.0–954.5)	0.0228^*^
**Centrally acting antihypertensive drugs**	***n*** **= 63**	***n*** **= 48**	***n*** **= 15**	
Daily dosage, mg/day	75.0 (25.0–524.7)	100.0 (27.81–524.7)	38.5 (25.0–556.46)	0.5393
Cumulative dosage, mg	525.0 (42.0–7945.0)	920.0 (79.5–7945.0)	175.0 (26.5–9175.0)	0.5291
Cumulative duration, days	135.0 (28.0–387.5)	139.5 (37.0–371.5)	28.0 (8.5–459.0)	0.4433
**Loop diuretics**	***n*** **= 1,636**	***n*** **= 1,042**	***n*** **= 594**	
Daily dosage, mg/day	27.39 (9.15–72.58)	27.76 (11.26–64.57)	25.66 (7.54–144.78)	0.5814
Cumulative dosage, mg	525.0 (76.0–2673.5)	577.0 (114.0–2721.75)	388.5 (48.0–2538.0)	0.0109^*^
Cumulative duration, days	158.0 (38.0–467.0)	184.0 (45.5–516.5)	128.0 (31.0–372.5)	0.0014^**^
**Phosphodiesterase type-3 inhibitors**	***n*** **= 13**	***n*** **= 7**	***n*** **= 6**	
Daily dosage, mg/day	7.7 (1.67–45.0)	16.0 (5.02–60.06)	2.97 (0.9–15.67)	0.1336
Cumulative dosage, mg	74.0 (3.84–171.0)	171.0 (15.05–296.88)	38.92 (2.73–104.5)	0.1336
Cumulative duration, days	3.0 (2.0–6.0)	3.0 (2.0–6.0)	4.5 (2.5–6.0)	0.9416
**Potassium-sparing diuretics and aldosterone**	***n*** **= 664**	***n*** **= 359**	***n*** **= 305**	
Daily dosage, mg/day	21.37 (8.09–29.17)	25.0 (12.5–35.69)	13.86 (6.36–25.0)	<0.0001^***^
Cumulative dosage, mg	568.0 (88.0–3676.88)	475.0 (75.0–4625.0)	654.0 (125.0–2975.0)	0.6552
Cumulative duration, days	98.5 (31.0–330.0)	87.0 (24.5–334.5)	117.0 (39.0–322.0)	0.1124
**Thiazides and related diuretics**	**n = 215**	***n*** **= 145**	***n*** **= 70**	
Daily dosage, mg/day	3.26 (1.77–9.65)	2.99 (1.67–5.62)	5.0 (2.5–17.08)	0.0322^*^
Cumulative dosage, mg	83.0 (15.0–531.25)	42.0 (7.0–231.0)	461.5 (70.75–922.0)	<0.0001^***^
Cumulative duration, days	43.0 (7.0–219.5)	21.0 (4.0–112.0)	116.0 (35.0–344.0)	<0.0001^***^
**Vasodilator antihypertensive drugs**	***n*** **= 346**	***n*** **= 202**	***n*** **= 144**	
Daily dosage, mg/day	35.48 (6.33–62.32)	39.22 (6.87–67.1)	33.85 (6.0–56.46)	0.0144^*^
Cumulative dosage, mg	474.0 (90.0-3040.5)	285.5 (73.0–1789.0)	966.0 (160.0–4419.0)	0.0033^**^
Cumulative duration, days	126.0 (22.5–419.0)	58.0 (14.0–269.0)	237.0 (42.5–724.5)	<0.0001^***^
**Laboratory examinations on PHTN**				
Hemoglobin, g/dL	12.1 (10.0–14.0); *n* = 2,515	11.6 (10.0–13.0); *n* = 1,340	12.4 (11.0–14.0); *n* = 1,175	0.0115^*^
Hematocrit, L/L	0.4 (0.0–0.0); *n* = 2,511	0.4 (0.0–0.0); *n* = 1,339	0.4 (0.0–0.0); *n* = 1,172	0.5656
Lymphocyte, x10∧9/L	1.3 (1.0–2.0); *n* = 2,506	1.0 (1.0–2.0); *n* = 1,339	1.7 (1.0–3.0); *n* = 1,167	0.1625
Neutrophil, x10∧9/L	5.3 (4.0–7.0); *n* = 2,506	5.4 (4.0–8.0); *n* = 1,339	5.0 (4.0–7.0); *n* = 1,167	0.0615
Platelet, x10∧9/L	197.0 (147.0–256.0); *n* = 2,508	178.0 (130.0–232.0); *n* = 1,336	221.5 (170.0–278.0); *n* = 1,172	<0.0001^***^
APTT, secs	32.9 (29.0–38.0); *n* = 2,352	33.3 (29.0–38.0); *n* = 1,299	32.7 (29.0–38.0); *n* = 1,053	0.0016^**^
INR	1.2 (1.0–1.0); *n* = 2,364	1.2 (1.0–2.0); *n* = 1,303	1.2 (1.0–1.0); *n* = 1,061	0.0166^*^
Prothrombin time, sec	13.4 (12.0–17.0); *n* = 2,333	13.8 (12.0–18.0); *n* = 1,292	13.1 (12.0–16.0); *n* = 1,041	0.6319
Red cell count, x10∧12/L	4.1 (4.0–5.0); *n* = 2,510	4.0 (3.0–5.0); *n* = 1,339	4.2 (4.0–5.0); *n* = 1,171	0.2716
Total protein, g/L	68.0 (62.0–74.0); *n* = 2,465	67.7 (62.0–73.0); *n* = 1,330	69.0 (62.0–75.0); *n* = 1,135	0.7112
Total bilirubin, umol/L	13.0 (8.0–22.0); *n* = 2,453	14.6 (9.0–24.0); *n* = 1,326	11.4 (7.0–20.0); *n* = 1,127	<0.0001^***^
Alkaline phosphatase, U/L	49.5 (34.0–73.0); *n* = 2,463	51.5 (35.0–74.0); *n* = 1,329	47.0 (32.0–72.0); *n* = 1,134	0.1625
Red cell distance width, %	15.1 (14.0–17.0); *n* = 2,472	15.7 (14.0–18.0); *n* = 1,317	14.5 (13.0–16.0); *n* = 1,155	0.0317^*^
Mean cell volume, fL	89.9 (85.0–95.0); *n* = 2,512	91.0 (86.0–96.0); *n* = 1,339	89.0 (84.0–93.0); *n* = 1,173	<0.0001^***^
Mean cell hemoglobin concentration, g/dL	34.2 (33.0–35.0); *n* = 2,512	34.2 (33.0–35.0); *n* = 1,339	34.3 (33.0–35.0); *n* = 1,173	0.0815

*APTT, activated partial thromboplastin time; INR, international normalized ratio; IQR, interquartile range; LOS, length of stay; PHTN, pulmonary hypertension*.

* * for P ≤ 0.05*,

** *for P ≤ 0.01*,

*** *for P ≤ 0.001*.

**Table 2:** The characteristics of patients who developed complications after the diagnosis of pulmonary hypertension.

**Characteristics**	**Cardiovascular complications (*n* = 1,878) Median (IQR) or count (%)**	**Renal complications (*n* = 684)** ** Median (IQR) or count (%)**	**Diabetes (*n* = 437) Median (IQR) or count (%)**	***P*-value**
**Demographics**				
Male sex	710 (37.80%)	283 (41.37%)	162 (37.07%)	0.0017^**^
Age at diagnosis	69.2 (48.0–81.0)	72.0 (54.0–82.0)	75.8 (66.0–83.0)	0.0008^***^
**Baseline hospitalizations**				
Total number of hospital admissions	15.0 (8.0–25.0)	19.0 (11.0–34.0)	19.0 (11.0–33.0)	<0.0001^***^
Number of emergency readmissions	2.0 (0.0–5.0)	3.0 (1.0–7.0)	2.0 (1.0–7.0)	0.0216^*^
Mean readmission interval (days)	211.3 (102.0–388.0)	188.0 (98.0–315.0)	219.1 (128.0–405.0)	<0.0001^***^
Cumulative length-of-stay	93.0 (44.0–170.0)	137.0 (74.0–236.0)	120.0 (63.0–226.0)	<0.0001^***^
**Baseline comorbidities**				
Respiratory disease	1,878 (100.00%)	684 (100.00%)	437 (100.00%)	0.5816
Hypertension	1,872 (99.68%)	682 (99.70%)	437 (100.00%)	0.2671
Cardiovascular disease	-	593 (86.69%)	382 (87.41%)	<0.0001^***^
Gastrointestinal disease	792 (42.17%)	377 (55.11%)	253 (57.89%)	0.0002^***^
Kidney disease	645 (34.34%)	-	242 (55.37%)	<0.0001^***^
Endocrine disease	56 (2.98%)	24 (3.50%)	14 (3.20%)	0.0017^**^
Diabetes mellitus	275 (14.64%)	190 (27.77%)	-	<0.0001^***^
Obesity	55 (2.92%)	30 (4.38%)	34 (7.78%)	<0.0001^***^
**Drug prescriptions for PHTN**				
Cardiac glycosides	466 (24.81%)	166 (24.26%)	98 (22.42%)	<0.0001^***^
Phosphodiesterase type-3 inhibitors	9 (0.47%)	5 (0.73%)	0 (0.00%)	0.0031^**^
Thiazides and related diuretics	190 (10.11%)	109 (15.93%)	73 (16.70%)	<0.0001^***^
Loop diuretics	1,377 (73.32%)	525 (76.75%)	323 (73.91%)	0.0211^*^
Potassium-sparing diuretics and aldosterone	579 (30.83%)	204 (29.82%)	108 (24.71%)	0.0022^**^
Anti-arrhythmias drugs	224 (11.92%)	98 (14.32%)	48 (10.98%)	<0.0001^***^
Beta blockers	567 (30.19%)	271 (39.61%)	192 (43.93%)	<0.0001^***^
Vasodilator antihypertensive drugs	232 (12.35%)	89 (13.01%)	44 (10.06%)	0.0012^**^
Centrally acting antihypertensive drugs	56 (2.98%)	30 (4.38%)	22 (5.03%)	<0.0001^***^
Alpha blockers	184 (9.79%); *n* = 184	116 (16.95%); *n* = 116	83 (18.99%); *n* = 83	<0.0001^***^
**Laboratory tests**				
Hemoglobin, g/dL	11.9 (10.0–14.0); *n* = 1,862	10.8 (9.0–13.0); *n* = 679	11.3 (10.0–13.0); *n* = 432	0.0035^**^
Hematocrit, L/L	0.4 (0.0–0.0); *n* = 1,861	0.3 (0.0–0.0); *n* = 679	0.3 (0.0–0.0); *n* = 432	0.6241
Lymphocyte, x10∧9/L	1.3 (1.0–2.0); *n* = 1,858	1.0 (1.0–2.0); *n* = 679	1.1 (1.0–2.0); *n* = 432	0.042^*^
Neutrophil, x10∧9/L	5.2 (4.0–7.0); *n* = 1,858	5.3 (4.0–7.0); *n* = 679	5.4 (4.0–7.0); *n* = 432	0.0071^**^
Platelet, x10∧9/L	191.5 (144.0–249.0); *n* = 1,858	180.5 (135.0–240.0); *n* = 678	191.0 (144.0–238.0); *n* = 431	0.0063^**^
APTT, secs	33.0 (30.0–38.0); *n* = 1,781	33.8 (30.0–39.0); *n* = 665	31.7 (28.0–36.0); *n* = 426	0.0163^*^
INR	1.2 (1.0–1.0); *n* = 1,789	1.2 (1.0–2.0); *n* = 668	1.2 (1.0–1.0); *n* = 425	0.0166^*^
Prothrombin time, sec	13.6 (12.0–17.0); *n* = 1,766	13.8 (12.0–17.0); *n* = 658	12.9 (12.0–15.0); *n* = 423	<0.0001^***^
Red cell count, x10∧12/L	4.1 (4.0–5.0); *n* = 1,861	3.8 (3.0–4.0); *n* = 679	4.0 (3.0–5.0); *n* = 432	0.0714
Total protein, g/L	68.0 (62.0–74.0); *n* = 1,831	68.0 (62.0–73.0); *n* = 671	70.0 (64.0–74.0); *n* = 430	0.5312
Total bilirubin, umol/L	14.0 (9.0–23.0); *n* = 1,834	13.0 (8.0–22.0); *n* = 668	11.4 (8.0–18.0); *n* = 431	<0.0001^***^
Alkaline phosphatase, U/L	49.0 (34.0–71.0); *n* = 1,826	53.6 (35.0–78.0); *n* = 668	48.5 (33.0–68.0); *n* = 431	<0.0001^***^
Red cell distribution width, %	15.2 (14.0–17.0); *n* = 1,844	15.7 (14.0–18.0); *n* = 670	15.0 (14.0–17.0); *n* = 431	0.0615
Mean cell volume, fL	90.1 (85.0–95.0); *n* = 1,861	90.5 (86.0–95.0); *n* = 679	89.8 (86.0–94.0); *n* = 432	<0.0001^***^
Mean cell hemoglobin concentration, g/dL	34.2 (33.0–35.0); *n* = 1,861	34.2 (33.0–35.0); *n* = 679	34.0 (33.0–35.0); *n* = 432	0.0915

*APTT, activated partial thromboplastin time; INR, international normalized ratio; IQR, interquartile range; LOS, length of stay; PHTN, pulmonary hypertension*.

* *for P ≤ 0.05*,

** *for P ≤ 0.01*,

*** *for P ≤ 0.001*.

### The Risk of Adverse Outcomes With Cox Analysis

**Figure 2:**
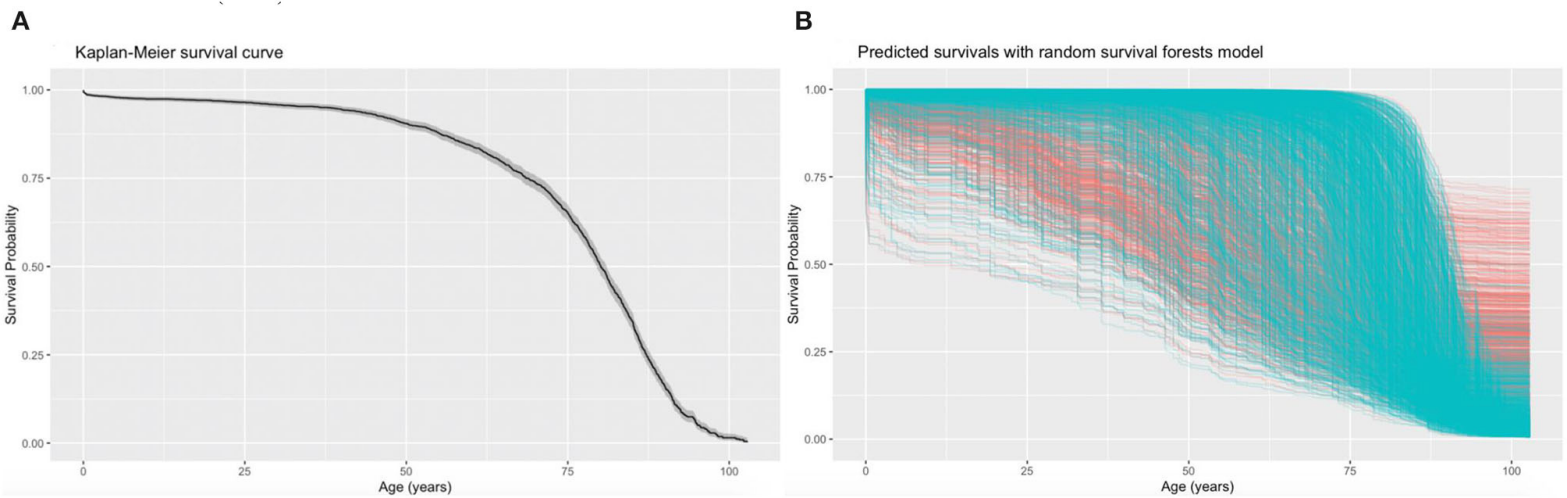
Kaplan-Meier curves for all-cause mortality and the predicted mortality. The Kaplan-Meier survival curves demonstrate the moralities of the patients with pulmonary hypertension (PHTN) **(A)** and the predicted mortalities using the random survival forest (RSF) prediction **(B)**. Each line represents a single patient in the training data set, where censored patients are colored blue, and patients who have experienced the mortality event are colored in red. The median survival (black) with a 95% shaded confidence band (gray) are indicated.

A univariable Cox regression analysis identified the predictors of mortality after the diagnosis of PHTN (Supplementary Table 3). Patients with past comorbidities, such as cardiovascular diseases (HR: 1.947, 95% CI: 0.8132–2.102), respiratory diseases (HR: 1.295, 95% CI: 0.646–2.595), endocrine disease (HR: 1.524, 95% CI: 1.127–2.059), and hypertension (HR: 1.362, 95% CI: 1.172–2.7618) were associated with higher mortality risks. The univariable Cox regression analysis also identified the predictors for the complications (Supplementary Table 4). The diagnosis age was an important predictor (all *P* < 0.0001). The multivariable Cox regression predictors of mortality were also identified (Supplementary Table 5A). The age of diagnosis (HR: 1.822; 95% CI: 1.815–1.829), cumulative hospital length of stay (LOS) (HR: 1.0007; 95% CI: 1.00040–1.0009), prior cardiovascular diseases (HR: 1.266; 95% CI: 1.064–1.507), kidney diseases (HR: 1.279; 95% CI: 1.125–1.454), diabetes mellitus (HR: 1.208; 95% CI: 1.032–1.415), and hypertension (HR: 1.549; 95% CI: 0.665–3.608) were predictive of mortality. The Kaplan-Meier survival curves demonstrated the mortality of the patients in Figure 2A.

### Machine Learning Survival Analysis

**Figure 3:**
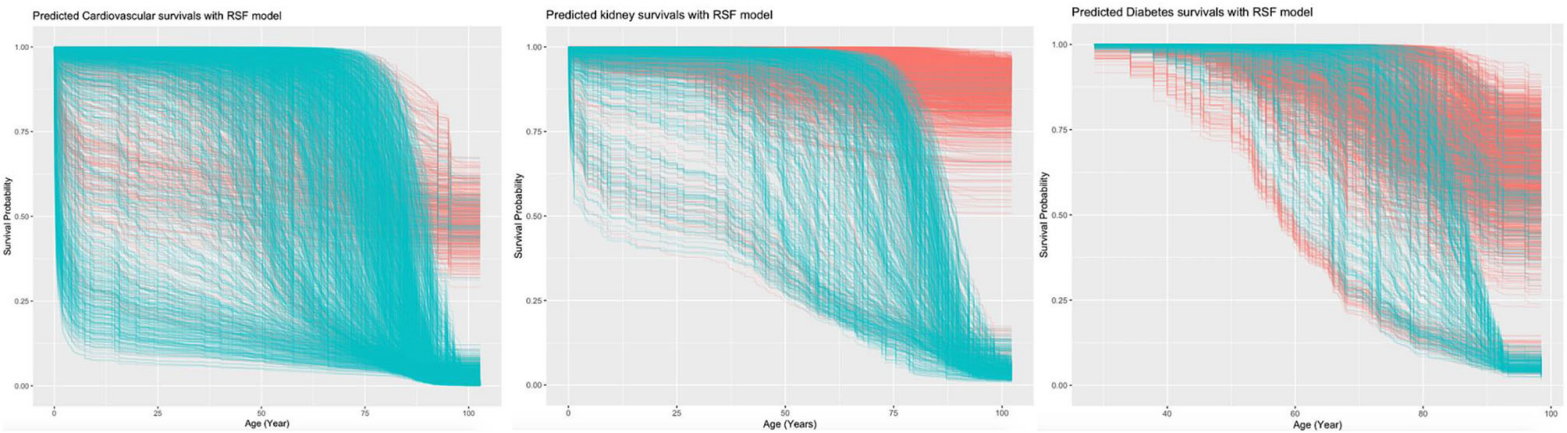
Predicted survivals of cardiovascular, kidney, and diabetes complications with RSF model.

The RSF model identified the influential prognostic risk factors by capturing the non-linearity and interactions. The predicted events for all-cause mortality with the RSF model are shown in Figure 2B. Most deaths occurred at age equal or above 75. The predictions for the time to event of the complications are shown in Figure 3. The RSF provided better discrimination performance in predicting the mortality risk, given its ability to capture the non-linearity and the interaction patterns (Supplementary Figures 4–7).

Age was the most important for the mortality risk prediction, followed by average readmission, antihypertension drugs, cumulative LOS, and total bilirubin level (Supplementary Figure 8). The risk factors that predicted the three complications were also identified. Age was the most important predictor for cardiovascular complications, followed by lymphocyte count and mean readmission interval. Age also showed the highest prediction strength for kidney and diabetic complications.

Important interactions with the demographics included the interactions of PHTN age and sex with average readmission interval, cumulative hospital stay, and total bilirubin level (Supplementary Figure 4). The interactions of the variables with past comorbidities (Supplementary Figure 5), drug prescriptions (Supplementary Figure 6), and laboratory examinations (Supplementary Figure 7) were also derived. An interaction importance ranking pattern could be observed: interactions formed by a variable with the influential individual risk factors demonstrated high predictive strength.

### Predictors of Adverse Events Risk With eFI

Correspondingly, an eFI was developed for predicting the all-cause mortality (Supplementary Table 5B). The calculated eFI for patients with/without mortality was significantly different (median: 9.0, IQR: 8.0–10.0 vs. median: 8.0, IQR: 6.0–9.0, *P* < 0.0001) (Table 3A, Supplementary Figure 9). The eFI was significantly associated with higher mortality risk (HR: 1.25, 95% CI: 1.22–1.29, *P* < 0.0001) (Table 3B). The marginal effects of eFI also demonstrated that higher eFI was associated with higher risks of mortality (Supplementary Figure 10).

**Table 3A:** Descriptive statistics of electronic frailty index for all-cause mortality risk prediction.

**Characteristics**	**Cut-off**	**All Median (IQR);**	**All-cause mortality Median (IQR);**	**Alive (*N* = 1,213)**	
		**(*N* = 2,560) N or Count (%)**	**(*N* = 1,347) N or Count (%)**	**Median (IQR); N or Count (%)**	***P*-value**
Electronic frailty index	9.5	8.0 (7.0–10.0); *n* = 2,560	9.0 (8.0–10.0); *n* = 1,394	8.0 (6.0–9.0); *n* = 1,166	<0.0001^***^
Electronic frailty index≥9.5	-	722 (28.20%)	527 (39.12%)	195 (16.08%)	<0.0001^***^

* *for P ≤ 0.05*,

** *for P ≤ 0.01*,

*** *for P ≤ 0.001*.

**Table 3B:** Prediction strength of electronic frailty index for all-cause mortality risk prediction.

**Characteristics**	**HR[CI]; *P*-value**	**Precision**	**Recall**	**AUC**	**C-index**
Electronic frailty index	1.25 [1.22–1.29]; <0.0001^***^	0.8234	0.8867	0.9015	0.9109
Electronic frailty index≥9.5	1.90 [1.70–2.12]; <0.0001^***^	0.8351	0.8909	0.9105	0.9202

* *for p ≤ 0.05*,

** *for p ≤ 0.01*,

*** *for p ≤ 0.001*.

**Figure 4:**
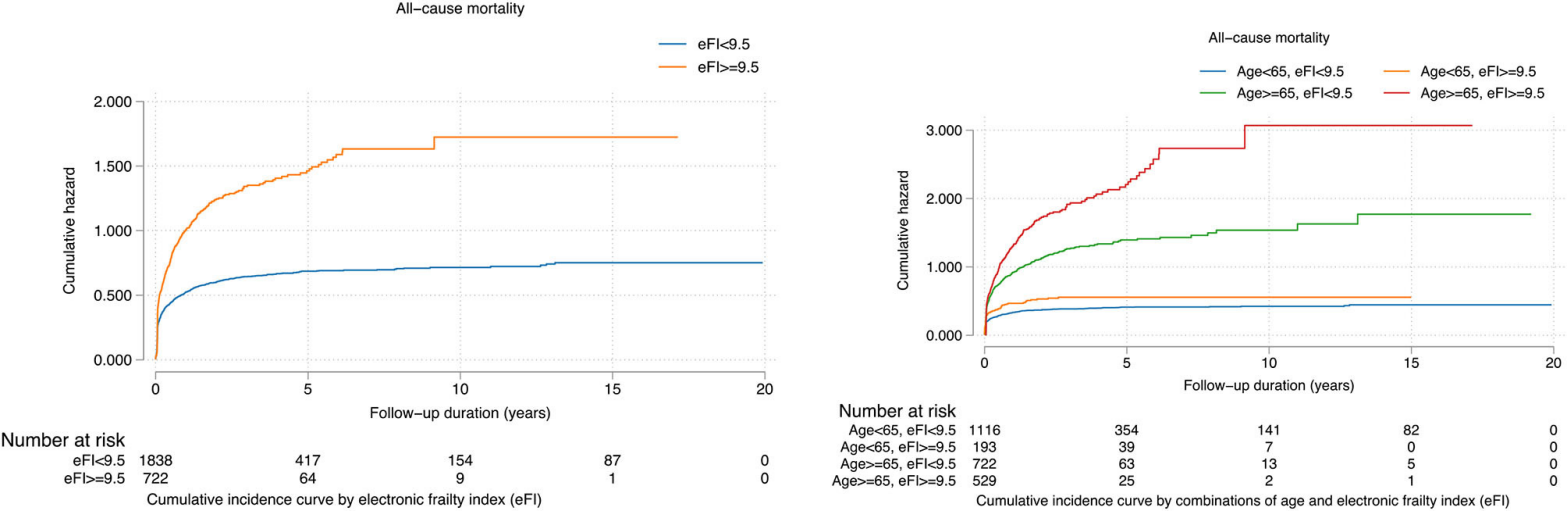
Cumulative incidence curves for all-cause mortality stratified by the constructed electronic frailty index and age.

The binarized eFI also predicted mortality risk based on the Youden cut-off of 9.5 (HR: 1.90, 95% CI: 1.70–2.12, *P* < 0.0001) (Table 3B). Patients with eFI > 9.5 had a higher cumulative hazard for all-cause mortality. The cumulative hazards were especially higher among patients who were 65 years old or above with eFI ≥ 9.5 (Figure 4). Within 2 years of follow-up, the eFI predicted the mortality (HR: 1.15, 95% CI: 1.02–1.28; *P* = 0.0169). The HR of the eFI increased with a longer duration of follow-up. Upon 10 years of follow-up, the eFI was associated with higher risks of mortality (HR: 1.71, 95% CI: 1.53–1.90; *P* < 0.0001); meanwhile, upon 20 years of follow-up, the HR was even higher (HR: 1.90; 95% CI: 1.70–2.12; *P* < 0.0001) (Table 3C).

**Table 3C:** Prediction strength of the constructed electronic frailty index for mortality risks within 2-year follow-up, 5-year follow-up, 10-year follow-up, 15-year follow-up, and 20-year follow-up.

**Characteristics**	**All-cause mortality HR [95% CI]; *P*-value**
**Within 2-year follow-up**	
Electronic frailty index	1.03 [1.00–1.07];0.0459^*^
<9	1.0 [Reference]
(9, 10)	1.15 [1.02–1.28];0.0183^*^
>12	1.15 [0.64–2.09];0.6394
High vs. low	1.15 [1.02–1.28];0.0169^*^
**Within 5-year follow-up**	
Electronic frailty index	1.15 [1.11–1.18]; <0.0001^***^
<9	1.0 [Reference]
(9, 10)	1.50 [1.34–1.67]; <0.0001^***^
>12	1.86 [1.03–3.38];0.0409^*^
High vs. low	1.51 [1.35–1.68]; <0.0001^***^
**Within 10-year follow-up**	
Electronic frailty index	1.21 [1.17–1.24]; <0.0001^***^
<9	1.0 [Reference]
(9, 10)	1.70 [1.52–1.90]; <0.0001^***^
>12	1.98 [1.09–3.59];0.0244^*^
High vs. low	1.71 [1.53–1.90]; <0.0001^***^
**Within 15-year follow-up**	
Electronic frailty index	1.22 [1.18–1.25]; <0.0001^***^
<9	1.0 [Reference]
(9, 10)	1.76 [1.58–1.96]; <0.0001^***^
>12	2.09 [1.15–3.79];0.0153^*^
High v.s. low	1.77 [1.58–1.97]; <0.0001^***^
**Within 20-year follow-up**	
Electronic frailty index	1.25 [1.22–1.29]; <0.0001^***^
<9	1.0 [Reference]
(9, 10)	1.89 [1.70–2.11]; <0.0001^***^
>12	2.23 [1.23–4.04];0.0085^**^
High vs. low	1.90 [1.70–2.12]; <0.0001^***^

* *for P ≤ 0.05*,

** *for P ≤ 0.01*,

*** *for P ≤ 0.001*.

*High: Electronic frailty index≥9.5; Low: Electronic frailty index <9.5*.

The RSF model showed a better performance in terms of precision (0.9263 vs. 0.8382), recall (0.9058 vs. 0.8992), AUC (0.9478 vs. 0.9051), and C index (0.9361 vs. 0.9240) compared to the Cox model in the 5-fold cross-validation. Similarly, compared to multivariate Cox regression, the precision, recall, AUC, and C index were significantly higher for RSF in predicting the cardiovascular, kidney, diabetic complications, and mortality (Table 4).

**Table 4A:** Performance comparisons between multivariable and random survival forest with five-fold cross validation.

**Model**	**Precision**	**Recall**	**AUC**	**C-index**
Multivariable Cox analysis	0.8382	0.8992	0.9051	0.9240
Random survival forests	0.9263	0.9058	0.9478	0.9361

**Table 4B:** Performance comparisons between multivariable Cox and random survival forest with five-fold cross validation.

	**Cardiovascular**		**Kidney**		**Diabetes**		**Mortality**	
**Metric**	**Multivariable Cox**	**RSF**	**Multivariable Cox**	**RSF**	**Multivariable Cox**	**RSF**	**Multivariable Cox**	**RSF**
Precision	0.84	0.92	0.83	0.92	0.84	0.92	0.84	0.93
Recall	0.83	0.91	0.78	0.91	0.87	0.91	0.83	0.91
AUC	0.90	0.95	0.85	0.94	0.85	0.95	0.76	0.94
C-index	0.88	0.94	0.87	0.93	0.90	0.94	0.79	0.91

## Discussion

### The Principal Findings of the Study

The main findings of this study include (i) risk factors including admission interval, cumulative LOS, and total admissions times were predictive of the complications and mortality; (ii) the RSF-identified non-linear relationship between the predictors and outcome was predictive of mortality; (iii) the RSF model performed better in mortality and complication predictions than the Cox regression; (iv) the eFI predicted the risks of all-cause mortality accurately, especially among patients who were 65 years old or above.

### Strength and Limitations of the Study

To the best of our knowledge, this is the first study using the eFI in predicting the PHTN outcomes. The usage of the cohort from a real-world clinical database to derive the RSF analysis was shown to have performed better than the multivariable logistic regression to predict the PHTN mortality and complications. This would allow better clinical management based on the eFI. However, there are certain limitations to this study. Firstly, given this is a local study conducted in Hong Kong, the PHTN results should be validated using the data from other databases in other countries. Secondly, medical history, such as smoking, asbestos, and family history, and clinical parameters such as partial pressure of oxygen (PaO2) and N-terminal pro-brain natriuretic peptide (NT-proBNP), which are associated with PHTN in other literature, were not included in this predictive model given the lack of the codes in CDARS (32). Thirdly, given the retrospective nature of this study, the results may be subjected to instabilities of the laboratory results, including the equipment modifications and blood samples artifacts, and changes in the clinical criteria for the diagnosis of the PHTN. Furthermore, clinical information suggested in the European PHTN guidelines, such as the clinical courses and syncope episodes, is also lacking. Furthermore, in our predictive model, the number of patients on centrally acting antihypertensive drugs and phosphodiesterase type-3 inhibitors was relatively small. Lastly, due to the lack of the relevant diagnostic codes, the PHTN cases were not classified according to the World Health Organization (WHO) PHTN classification (2). Nevertheless, frailty models with the lack of enlisted information are still strong predictors of mortality and mortality (33).

### Comparing the Findings With the Other Studies

Our data presented different risk factors that contributed to the development of the PHTN complications. Older patients and male patients showed a higher mortality risk. Patients with a higher comorbidity burden, including cardiovascular, hypertension, and renal conditions, had a lower survival rate and were more likely to develop complications (34). The variable importance and minimal depth approach indicated that the age of PHTN diagnosis had the highest predictive strength. This is in accordance with the previous predictive model (REVEAL), indicating that old male patients would have a worse prognosis, even though PHTN is a predominantly female disorder. This is worrying given the average age of patients diagnosed is shifting toward an older population (35). In agreement with previous studies, the hospitalization characteristics and drugs are also associated with the survival outcome in a pattern (36, 37). Compared to other predictive models, our study does not involve the use of the hemodynamic data nor lung function and radiological test results (38). However, our study demonstrated a higher AUC compared to the previous studies (39, 40). Nevertheless, the PHTN diagnosis in our study was not classified according to the WHO classification owing to the lack of the CDARS code.

Our results demonstrated that eFI significantly predicts the risks of complications and mortality among elderlies. Compare to the traditional Cox models, prediction models based on RSF have improved prediction performance across diseases such as heart failure (41), Brugada syndrome (42), congenital (43, 44) and acquired (45) long QT syndromes, diabetes mellitus (46, 47), metabolic diseases (48), stroke (49), and cancer (50). It improved the risk prediction in the context of PHTN for disease onset (51) and pressure prediction based on echocardiographic parameters (52). However, few studies have examined the long-term prognosis of patients with PHTN. In our study, the RSF model had a higher predictive accuracy with 5-fold cross-validation than the Cox model since it does not have a strong assumption about individual proportional hazard functions. Furthermore, the model can capture the interactions, reducing the prediction variances and bias (20, 53). The interactions formed by a variable with the influential individual risk factors demonstrated a high predictive strength.

### The Implications for the Clinicians and Future Research Directions

The eFI derived from the significant variables allows predicting the risks of mortality and complications of the patients with PHTN, especially among elderlies. Clinicians can make use of the electronic medical records to estimate the outcome of the patients with PHTN without using any hemodynamic and radiological investigation modalities. The eFI can be further translated into a risk diagnosis tool to be deployed in the computer for real-time clinical applications.

## Conclusion

The RSF model identified the influential prognostic risk factors and their interaction from the clinical accessible data. The usage of this RSF-derived eFI would allow stratifying the risks of complication and mortality and optimizing the PHTN management among elderlies.

## Data Availability Statement

The original contributions presented in the study are included in the article/Supplementary Material, further inquiries can be directed to the corresponding author/s.

## Ethics Statement

The studies involving human participants were reviewed and approved by NTEC-CUHK, Hong Kong. The Ethics Committee waived the requirement of written informed consent for participation.

## Author Contributions

OC, JZ, and SL: data analysis, data interpretation, statistical analysis, manuscript drafting, and critical revision of manuscript. KW, KL, IW, TL, and BC: project planning, data acquisition, data interpretation, and critical revision of manuscript. QZ and GT: study conception, study supervision, project planning, data interpretation, statistical analysis, manuscript drafting, and critical revision of manuscript. All authors contributed to the article and approved the submitted version.

## Funding

QZ acknowledges the National Key Research and Development Program of China, Ministry of Science and Technology of China: 2019YFE0198600; National Natural Science Foundation of China (NSFC): 71972164 and 72042018; Health and Medical Research Fund of the Food and Health Bureau of Hong Kong: 16171991; Innovation and Technology Fund of Innovation and Technology Commission of Hong Kong: MHP/081/19.

## Conflict of Interest

The authors declare that the research was conducted in the absence of any commercial or financial relationships that could be construed as a potential conflict of interest.

## Publisher's Note

All claims expressed in this article are solely those of the authors and do not necessarily represent those of their affiliated organizations, or those of the publisher, the editors and the reviewers. Any product that may be evaluated in this article, or claim that may be made by its manufacturer, is not guaranteed or endorsed by the publisher.
