# Supplementary material for: Development of an Electronic Frailty Index for Predicting Mortality and Complications Analysis in Pulmonary Hypertension Using Random Survival Forest Model
Source: Front Cardiovasc Med. 2022 Jul 8;9:735906. doi: 10.3389/fcvm.2022.735906 (PMC9304657; doi:10.3389/fcvm.2022.735906)
Supplement: Supplementary file 1 [file Data_Sheet_1.docx]

**Supplementary Table 1. Codes for comorbidities of Pulmonary Hypertension Patients**

| **Comorbidity** | **Codes** |
| --- | --- |
| Cardiovascular | 427.31, 423.9, 427.5, 428, 785.1, 413.9, 427.89, 410.71, 427.31, 423.9, 785, 794.31, 428.9, 427.89, 427.89, 427.81, 427.69, 427.32, 427, 426.4, 426.11, 426.1, 425.4, 424.9, 424, 421, 398.9, 787.01, 151, 414.9, V72.81, 410.01, V43.3, 427.5, 164.1, 402.9, 427.89, 785.51, 426, 414.8, 425.4, 414.9, 428.1, 309, 402.91, 404.93, 746.87, V81.0, 414, 648.63, 296.53, 300.4, 411, 426.13, 528.3, 996.2, 416.9, 429.3, 787.1, 785.9, 427.2, 786.51, 414.1, 398.91, 426.12, 391.9, 404.11, 416.9, 416, 416.8, 426, 746.89, 745.8, 746, 746.86, 746.9, 745.9, 648.5, E878.0, 746.9, 648.51, V53.31, 404.9, 746.7, 746.89, 404.91, 404.92, 428.1, 404.01, 648.53, V71.7, 398, 404.93, 397.9, 398.9, 429.3, 426.13, E879.0, 410, 410.9, 745.6, 427.89, 427, 648.61, 427.89, 745.6, 397, 429.4, 427.89, 443.9, 425.4, 427.89, 412, 423, 427, 423.9, V45.00, 972, 427.5, 427.89, 414.8, 427.89, 427.81, 410.71, 427.5, 785.2, 427.1, 745.69, 426.1, 431, 429.3, 427.69, 427.89, 996.63 |
| Respiratory | 786.09, 518.81, 780.53, 137, E912, 465.9, 518.81, 518.81, 79.6, 518.81, 519.8, 780.59, 799.1, 780.57, 518.82, 480.1, 786.3, 519.8, 997.3, 165.9, 519.9, 648.91, 162.9, 162.3, 197, 162.5, 162.4, 486, 518.89, 496, 162.8, 415.1, V10.11, 518, 162.9, 11.96, 482.1, 507, 513, 11.9, 511.8, 511.1, 11.94, 516.8, 793.1, 482.4, 507, 515, 197, 11.93, 482, 482.83, 518.4, 482.3, 482.2, 415.1, 502, 518.89, 235.7, 793.1, 934.8, 516.9, 136.3, 38.49, 506, 112.4, 487, 481, 117.9, 38.2, 518, 11.95, 79.89, 518.1, 480.9, 505, 516.8, 495.9, 518.3, 11.23, 416.8, 513, 397.1, 117.3, 483, 508, 998.81, 416, 514, 861.21, 502, 934.8, 480.8, 648.93, 11.2, 492.8, 484.6, 78.5, 484.1, 516.3, 415.1, 416.9, 415, 416.9, 429.89, 415, 747.49, 745, 417, 770.7, 427.5, 416.9, 416, 416.8, 746.02, 573.8, 642.9, 416, 747.3, 747.3, 770.3, 779.8, 515, 424.3, 416, 417.8, 747.3, 747.3, 745.4, 518.81, 786.09, V12.6, 478, 748.5, 162.9, 996.84, 748.5, 748.6, V42.1, 748.5, 11.05, 162, 518, 747.42, 518.89, 748.5, 517.2 |
| Kidney | 198.7, 189, 189, 585.9, V56.0, 189.1, 584.9, 189.1, 593.9, 189, 189, 189.8, 239.5, 189, 583.81, 593.9, 591, V10.52, 250.4, 591, 255.4, 590.8, 586, 585.1, 591, 189, 591, 239.7, 788, 996.39, 189.1, 588.9, 592, 996.39, 250.4, 593.2, 255.4, 572.4, 194, 255, 453.3, 198, 585.9, 996.39, 996.39, 590.1, 591, 591, 223, 591, 584.9, 585.9, 250.41, 996.39, 581.9, 227, 593.5, 583.89, 593.89, 404.93, 255.5, 788.9, 250.43, 227, 405.92, 592, 753.12, 996.39, E879.1, V42.0, 592, 580.89, 403.9, 593.9, V59.4, 585.9, 580.9, 250.41, 996.39, 585.9, 794.4, 584.8, 584.5, 255.9, 441.4, V58.49, 404.11, 593.9, 592, 585, 759.1, 753.11, E879.1, 753.15, 585.9, 274, 588.8, 403.91, 404.9, 404.91, 404.92, 403, 404.01, 779.8, 250.4, 227, 227, 227, 404.93, 996.81, 593.89, 753.8, 593.2, 592, 753, 996.81, 223, 589.1, 996.81, 582.9, 585.9, 996.81, 753.1, 753.3, E878.0, 593.9, 753.3, 753.17, 583.9, 593.9, 589, 866 |
| Endocrine | 202.8, 200.1, 200.12, 201.9, 204, 202.88, 200.18, 196, 204.01, 785.6, 200.11, 200.13, 202.8, 785.6, 202.85, 202.81, 202.8, 202.8, 196, 196.9, 202.8, 202.82, 785.6, 202.8, 202, 202.87, V10.79, 785.6, 202.84, 202.01, 196.8, 457.1, 12.1, 785.6, V10.79, 196.5, 196.2, 238.7, 196.1, 200.14, 457.2, 238.7, V10.61, 201.9, 457, 202.8, 289.3, 245.2, 238.7, 785.6, 457.9, 785.6, 202.93, 196.9, 202.97, 757, V10.71, 288.8, 204.1, 202.83, 457.1, 289.3, 785.6, V77.9, 237.4, 239.7, 198.89, 623.5, 259.9, 200.2, V10.71 |
| Diabetes mellitus | 251.2, 362.01, 362.02, 250.4, 250.82, 790.2, 790.6, 250.5, 250.5, 250.6, 357.2, 790.2, 250, 250.4, 250.6, 250.8, 250.51, 250.5, 250.8, 250.82, 250.51, 250.51, V77.1, 250.12, 251.2, 250.12, 250.5, 250.83, 251.2, 250.41, 251.1, 250.52, 250.5, 648.81, 250.43, 250.53, 250.53, 250.81, 250.22, 250.13, 250.22, 250.83, 250.41, 250.5, 250.52, 250.52, 250.82, 253.5, V18.0, 588.1 |
| Hypertension | 401.9, 401.9, 250.82, 790.6, 401.9, 401.9, 250.82, 796.2, 402.9, 250.83, 405.99, 642.93, 642.01, 642.91, 401.9, E942.6, 405.09, 403.9, 437.2, 401, 401.1, 401, 642.33, 348.2, 779.8, 365.04, 572.3, 416, 416, 405.91, 416.8, 642.3 |
| Gastrointestinal | 153.3, 154.1, 153.9, 569.89, 154, 153.1, 578.9, 560.9, 569.3, 537.89, 558.9, 562.1, 153.6, 239, 532.3, 569.89, 532.7, 535.6, 558.9, 38.42, 569.89, 8.45, 153.2, 569.49, 79.89, 532.9, V58.11, 569, 154.1, 41.4, 537.89, 152.1, 578.9, V10.05, 787.8, 197.4, 535.5, V10.06, 9, 569.83, 569.6, 153.4, 560.9, 537.3, 41.04, 569.84, 239, 569.81, 8.8, 535, 560.9, 532, V45.89, V12.72, 532.4, V10.09, 560.81, 235.2, 38.49, 8.45, 235.2, 532.9, 569.81, 537.89, 557.9, 569.41, 997.4, 14.8, 787.99, 8.46, 535.5, 569.41, 997.4, 578.9, 569.82, 537.9, 560.1, 569.82, 557.9, 211.3, 556.9, 562, 558.9, 578.9, 536.9, 8.46, 535.6, 566, V71.9, 569.49, 564.3, V44.4, 569.89, 564.8, 8.46, 569.83, 997.4, 997.4, 997.4, 562.11, 211.2, 9.1, 211.3, 8.47, 8.5, 211.3, 569.83, 532.1, 535.61, 560, 569.83, 565.1, 619.1, 152.9, 568, 566, 569.43, 152, 8.46, 562.11, 8.61, 569.83, 569.83, 569.81, 596.1, 535.5, 151.4, 151.9, 151.5, 151.8, 151.1, 456.8, 531.7, 535.4, 531.3, V15.2, 537.89, 211.1, 531.9, V10.04, 235.2, 531, 531.4, 456.8, 535.1, 151.3, 230.2, 151.6, 535, 211.1, 536.3, 535, V10.04, 535.51, 578.9, 531.1, 535.1, 456.8, 531.5, 537.84, 535.01, 530.7, 535.1, 535.2, 535.5, 537.6, 202.83, 535.1, 531.4, 558.9, 558.9, 558, 569.85, 153, 555.1, 562.1, 562.13, 562.11, 562.12, 569.83, V76.49, 560.2, 230.4, 569.3, 154 |
| Obesity | 278.01, 278.00, 278, |

**Supplementary Table 2. Drugs in each drug categories for pulmonary hypertension treatment (prescribed for the study cohort)**

| **Drug Therapy** | **Drugs** |
| --- | --- |
| Cardiac glycosides | DIGOXIN, METILDIGOXIN |
| Phosphodiesterase type-3 inhibitors | MILRINONE |
| Thiazides and related diuretics | BENDROFLUAZIDE, HYDROCHLOROTHIAZIDE, INDAPAMIDE, METHYCLOTHIAZIDE, METOLAZONE |
| Loop diuretics | BUMETANIDE, FRUSEMIDE |
| Potassium-sparing diuretics and aldosterone | AMILORIDE, EPLERENONE, SPIRONOLACTONE, DYAZIDE, MODURETIC, NITROPRUSSIDE |
| Anti-arrhythmias drugs | AMIODARONE, ATROPINE, DISOPYRAMIDE, DRONEDARONE, FLECAINIDE, MEXILETINE, PROPAFENONE, QUINIDINE |
| Beta blockers | ATENOLOL, CARVEDILOL, CELIPROLOL, ESMOLOL, LABETALOL, METOPROLOL, NADOLOL, NEBIVOLOL, PINDOLOL, PROPRANOLOL, SOTALOL |
| Vasodilator antihypertensive drugs | BOSENTAN, DIHYDRALAZINE, DIHYDRALAZINE, ILOPROST, MACITENTAN, METARAMINOL, SILDENAFIL, TOLAZOLINE |
| Centrally acting antihypertensive drugs | CLONIDINE, METHYLDOPA |
| Alpha blockers | PHENOXYBENZAMINE, DOXAZOSIN, PRAZOSIN, TERAZOSIN |

**Supplementary Table 3. Univariable Cox predictors of all-cause mortality outcome**

APTT: Activated partial thromboplastin time; INR: International normalized ratio; IQR: Interquartile range; LOS: Length of stay; PHTN: Pulmonary hypertension.

* for p≤ 0.05, ** for p ≤ 0.01, *** for p ≤ 0.001

| **Characteristics** | **Hazard ratio (95% CI)** | **Z value** | **P value** |
| --- | --- | --- | --- |
| **Demographics** |  |  |  |
| Male sex | 1.209(1.083,1.351) | 3.371 | 0.000748*** |
| Age at diagnosis | 1.8603(1.8545,1.8662) | -43.23 | <2e-16*** |
| **Hospitalization** |  |  |  |
| Total number of hospital admissions | 1.001(1.001,1.002) | 6.036 | 1.58E-09*** |
| Number of emergency readmissions | 1.011(1.004,1.018) | 3.309 | 0.000937*** |
| Mean readmission interval (days) | 0.9997(0.9996,0.9999) | -3.594 | 0.000325*** |
| Cumulative length-of-stay | 1.001(1.001,1.001) | 9.683 | <2e-16*** |
| **Comorbidity** |  |  |  |
| Cardiovascular disease | 1.9468(0.8132,2.102) | -0.704 | 0.00013*** |
| Respiratory disease | 1.295(0.646,2.595) | 0.728 | 0.000025*** |
| Kidney disease | 1.077(0.9656,1.202) | 1.333 | 0.183 |
| Endocrine disease | 1.524(1.127,2.059) | 2.739 | 0.00617** |
| Diabetes mellitus | 1.008(0.8717,1.166) | 0.108 | 0.914 |
| Hypertension | 1.362(1.172,2.7618) | -2.677 | 0.00743** |
| Gastrointestinal disease | 0.8429(0.7568,0.9387) | -3.11 | 0.00187** |
| Obesity | 0.7841(0.5426,1.133) | -1.295 | 0.195 |
| **Drug prescriptions (mean daily dosage)** |  |  |  |
| Cardiac glycosides | 1.079(0.9566,1.218) | 1.24 | 0.215 |
| Phosphodiesterase type-3 inhibitors | 21.36(9.92,46.01) | 7.823 | 5.18E-15*** |
| Thiazides and related diuretics | 1.114(0.9375,1.324) | 1.227 | 0.22 |
| Loop diuretics | 1.08(0.9501,1.228) | 1.178 | 0.239 |
| Potassium-sparing diuretics and aldosterone | 1.372(1.216,1.549) | 5.131 | 2.88E-07*** |
| Anti-arrhythmias drugs | 1.205(1.029,1.411) | 2.316 | 0.0206* |
| Beta blockers | 0.7919(0.7046,0.8901) | -3.911 | 9.18E-05*** |
| Vasodilator antihypertensive drugs | 2.65(2.274,3.09) | 12.46 | <2e-16*** |
| Centrally acting antihypertensive drugs | 0.7883(0.5908,1.052) | -1.617 | 0.106 |
| Alpha blockers | 0.7782(0.6592,0.9187) | -2.962 | 0.00306** |
| **Laboratory examinations** |  |  |  |
| Hemoglobin, g/dL | 1.047(1.024,1.07) | 4.081 | 4.49E-05*** |
| Alkaline phosphatase, U/L | 1.005(1.004,1.006) | 11.08 | <2e-16*** |
| Hematocrit, L/L | 4.913(2.314,10.43) | 4.144 | 3.42E-05*** |
| Lymphocyte, x10^9/L | 1.054(1.001,1.111) | 1.99 | 0.0465* |
| Neutrophil, x10^9/L | 1.049(1.036,1.061) | 7.804 | 6.02E-15*** |
| Platelet, x10^9/L | 0.9994(0.9987,1) | -1.728 | 0.084. |
| APTT, secs | 1.018(1.014,1.021) | 9.541 | <2e-16*** |
| INR | 1.502(1.393,1.619) | 10.57 | <2e-16*** |
| Prothrombin time, sec | 1.033(1.026,1.039) | 10.29 | <2e-16*** |
| Red cell count, x10^12/L | 1.12(1.053,1.192) | 3.61 | 0.000306*** |
| Total protein, g/L | 0.997(0.9925,1.001) | -1.32 | 0.187 |
| Total bilirubin, umol/L | 1.004(1.003,1.005) | 8.91 | <2e-16*** |
| Red cell distance width, % | 1.068(1.05,1.087) | 7.359 | 1.85E-13*** |
| Mean cell volume, fL | 1.005(1.001,1.009) | 2.295 | 0.0217* |
| Mean cell hemoglobin concentration, g/dL | 1.014(0.9997,1.028) | 1.919 | 0.055. |

**Supplementary Table 4. Univariable Cox predictors of cardiovascular, renal and diabetes outcomes**

APTT: Activated partial thromboplastin time; HR: Hazard ratio; INR: International normalized ratio; IQR: Interquartile range; LOS: Length of stay; PHTN: Pulmonary hypertension.

* for p≤ 0.05, ** for p ≤ 0.01, *** for p ≤ 0.001

|  | **Cardiovascular (n=1878)** | | | **Kidney (n=684)** | | | **Diabetes (n=437)** | | |
| --- | --- | --- | --- | --- | --- | --- | --- | --- | --- |
| **Characteristics** | **Z value** | **P value** | **HR (95% CI)** | **Z value** | **P value** | **HR (95% CI)** | **Z value** | **P value** | **HR (95% CI)** |
| **Demographics** |  |  |  |  |  |  |  |  |  |
| Male sex | 3.14 | 0.00169** | 1.16 [1.06 ,1.28] | 4.07 | <0.0001*** | 1.37 [1.18 ,1.60] | 1.34 | 0.181 | 1.14 [0.94 ,1.39] |
| Age at diagnosis | -55.01 | <0.0001*** | 1.84 [1.83 ,1.84] | -31.03 | <0.0001*** | 1.86 [1.85 ,1.87] | -25.52 | <0.0001*** | 1.86 [1.85 ,1.87] |
| **Hospitalization before PHTN** |  |  |  |  |  |  |  |  |  |
| Total number of hospital admissions | 3.33 | 0.0009*** | 1.00 [1.00 ,1.00] | 10.25 | <0.0001*** | 1.00 [1.00 ,1.00] | 4.83 | <0.0001*** | 1.00 [1.00 ,1.00] |
| Number of emergency readmissions | -1.30 | 0.192 | 1.00 [0.99 ,1.00] | 9.50 | <0.0001*** | 1.03 [1.02 ,1.03] | 4.19 | <0.0001*** | 1.02 [1.01 ,1.03] |
| Mean readmission interval (days) | -4.65 | <0.0001*** | 1.00 [1.00 ,1.00] | -5.28 | <0.0001*** | 1.00 [1.00 ,1.00] | -2.14 | 0.0324* | 1.00 [1.00 ,1.00] |
| Cumulative length-of-stay | 3.49 | 0.0005*** | 1.00 [1.00 ,1.00] | 13.03 | <0.0001*** | 1.00 [1.00 ,1.00] | 8.83 | <2e-16*** | 1.00 [1.00 ,1.00] |
| **Comorbidities before PHTN** |  |  |  |  |  |  |  |  |  |
| Cardiovascular disease | - | - | - | 1.04 | 0.301 | 1.12 [0.90 ,1.40] | 0.07 | 0.942 | 1.01 [0.76 ,1.34] |
| Respiratory disease | - | - | - | - | - | - | - | - | - |
| Kidney disease | -3.03 | 0.0024** | 0.86 [0.78 ,0.95] | - | - | - | 7.10 | <0.0001*** | 1.98 [1.64 ,2.39] |
| Endocrine disease | 1.73 | 0.0844. | 1.27 [0.97 ,1.65] | 2.10 | 0.0354* | 1.55 [1.03 ,2.33] | 1.73 | 0.0834. | 1.60 [0.94 ,2.73] |
| Diabetes mellitus | -1.87 | 0.0609. | 0.88 [0.78 ,1.01] | 8.09 | <0.0001*** | 2.00 [1.69 ,2.37] | - | - | - |
| Hypertension | -0.82 | 0.411 | 0.71 [0.32 ,1.59] | -0.44 | 0.661 | 0.73 [0.18 ,2.94] | 0.01 | 0.991 | 0.78 [0.13 ,1.24] |
| Gastrointestinal disease | -5.51 | <0.0001*** | 0.77 [0.70 ,0.85] | 3.46 | 0.0006*** | 1.31 [1.12 ,1.52] | 3.35 | 0.0008*** | 1.39 [1.15 ,1.68] |
| Obesity | 0.07 | 0.948 | 1.01 [0.77 ,1.32] | 2.43 | 0.015* | 1.58 [1.09 ,2.27] | 5.98 | 2.23E-09*** | 2.92 [2.05 ,4.14] |
| **Drug prescriptions after PHTN** |  |  |  |  |  |  |  |  |  |
| Cardiac glycosides | -0.35 | 0.73 | 0.98 [0.88 ,1.09] | -0.48 | 0.635 | 0.96 [0.80 ,1.14] | -1.81 | 0.071. | 0.81 [0.65 ,1.02] |
| Phosphodiesterase type-3 inhibitors | 5.81 | <0.0001*** | 7.12 [3.67 ,13.80] | 6.36 | <0.0001*** | 18.94 [7.66 ,46.86] | -0.01 | 0.992 | 3.23 [1.67 ,6.26] |
| Thiazides and related diuretics | 0.60 | 0.551 | 1.05 [0.90 ,1.22] | 5.44 | <0.0001*** | 1.77 [1.44 ,2.17] | 4.71 | <0.0001*** | 1.83 [1.42 ,2.35] |
| Loop diuretics | -1.45 | 0.148 | 0.93 [0.84 ,1.03] | 0.93 | 0.354 | 1.09 [0.91 ,1.30] | -1.66 | 0.0966. | 0.83 [0.67 ,1.03] |
| Potassium-sparing diuretics and aldosterone | -1.45 | 0.148 | 0.93 [0.84 ,1.03] | 0.93 | 0.354 | 1.09 [0.91 ,1.30] | -1.66 | 0.0966. | 0.83 [0.67 ,1.03] |
| Anti-arrhythmias drugs | 0.83 | 0.408 | 1.06 [0.92 ,1.22] | 2.57 | 0.0103* | 1.32 [1.07 ,1.64] | -0.30 | 0.767 | 0.96 [0.71 ,1.29] |
| Beta blockers | -3.66 | 0.0003*** | 0.83 [0.75 ,0.92] | 2.77 | 0.0056** | 1.24 [1.07 ,1.45] | 3.68 | 0.0002*** | 1.43 [1.18 ,1.72] |
| Vasodilator antihypertensive drugs | 7.80 | <0.0001*** | 1.75 [1.52 ,2.01] | 5.93 | <0.0001*** | 1.99 [1.59 ,2.50] | 3.65 | 0.0003*** | 1.80 [1.31 ,2.47] |
| Centrally acting antihypertensive drugs | -2.74 | 0.0061** | 0.69 [0.53 ,0.90] | -0.02 | 0.984 | 1.00 [0.69 ,1.44] | 0.40 | 0.692 | 1.09 [0.71 ,1.68] |
| Alpha blockers | -5.27 | <0.0001*** | 0.66 [0.57, 0.77] | 1.94 | 0.0518. | 1.22 [1.00 ,1.49] | 2.10 | 0.0356* | 1.29 [1.02 ,1.64] |
| **Laboratory examinations on PHTN** |  |  |  |  |  |  |  |  |  |
| Hemoglobin, g/dL | 7.454 | <0.0001*** | 1.08[1.058, 1.102] | -5.397 | <0.0001*** | 0.91[0.88, 0.94] | -0.863 | 0.388 | 0.98[0.94, 1.025] |
| Alkaline phosphatase, U/L | 9.254 | <0.0001*** | 1.004[1.003, 1.005] | 8.974 | <0.0001*** | 1.005[1.004,1.006] | 2.416 | 0.0157* | 1.003[1.001,1.01] |
| Hematocrit, L/L | 6.612 | <0.0001*** | 10.67[5.29,21.53] | -5.964 | <0.0001*** | 0.02[0.007, 0.08] | -0.988 | 0.323 | 0.47[0.10,2.12] |
| Lymphocyte, x10^9/L | 17.17 | <0.0001*** | 1.21 [1.18 ,1.24] | 1.927 | 0.054. | 1.07 [1.00 ,1.14] | 3.591 | 0.0003*** | 1.13 [1.06 ,1.20] |
| Neutrophil, x10^9/L | 2.641 | 0.00827** | 1.02 [1.01 ,1.03] | 1.887 | 0.0592. | 1.02 [1.00 ,1.04] | 1.509 | 0.131 | 1.02 [0.99 ,1.05] |
| Platelet, x10^9/L | 4.938 | <0.0001*** | 1.00 [1.00 ,1.00] | -0.128 | 0.898 | 1.00 [1.00 ,1.00] | 1.066 | 0.286 | 1.00 [1.00 ,1.00] |
| APTT, secs | 8.832 | <0.0001*** | 1.02 [1.01 ,1.02] | 7.793 | <0.0001*** | 1.02 [1.02 ,1.03] | -0.14 | 0.889 | 1.00 [0.99 ,1.01] |
| INR | 7.17 | <0.0001*** | 1.32 [1.22 ,1.42] | 4.239 | <0.0001*** | 1.31 [1.16 ,1.49] | -0.384 | 0.701 | 0.96 [0.78 ,1.18] |
| Prothrombin time, sec | 7.425 | <0.0001*** | 1.02 [1.02 ,1.03] | 4.188 | <0.0001*** | 1.02 [1.01 ,1.03] | -0.212 | 0.832 | 1.00 [0.98 ,1.02] |
| Red cell count, x10^12/L | 6.886 | <0.0001*** | 1.22 [1.15 ,1.29] | -5.744 | <0.0001*** | 0.75 [0.68 ,0.82] | 0.525 | 0.599 | 1.03 [0.91 ,1.17] |
| Total protein, g/L | -1.79 | 0.0734. | 0.99 [0.99 ,1.00] | -2.825 | 0.0047** | 0.99 [0.98 ,1.00] | 4.442 | <0.0001*** | 1.03 [1.02 ,1.04] |
| Total bilirubin, umol/L | 7.741 | <0.0001*** | 1.00 [1.00 ,1.01] | 3.17 | 0.0015** | 1.00 [1.00 ,1.01] | -0.72 | 0.471 | 1.00 [0.99 ,1.00] |
| Red cell distance width, % | 3.029 | 0.0025** | 1.03 [1.01 ,1.05] | 7.433 | <0.0001*** | 1.10 [1.08 ,1.13] | -0.06 | 0.952 | 1.00 [0.96 ,1.04] |
| Mean cell volume, fL | -1.811 | 0.0702. | 1.00 [0.99 ,1.00] | -0.064 | 0.949 | 1.00 [0.99 ,1.01] | -2.767 | 0.0056** | 0.99 [0.98 ,1.00] |
| Mean cell hemoglobin concentration, g/dL | 2.129 | 0.0332* | 1.02 [1.00 ,1.04] | 0.573 | 0.567 | 1.01 [0.98 ,1.04] | -1.353 | 0.176 | 0.97 [0.94 ,1.01] |

**Supplementary Table 5A. Multivariable Cox predictors of all-cause mortality outcome**

APTT: Activated partial thromboplastin time; INR: International normalized ratio; IQR: Interquartile range; LOS: Length of stay; PHTN: Pulmonary hypertension.

* for p≤ 0.05, ** for p ≤ 0.01, *** for p ≤ 0.001

| **Characteristics** | **Hazard ratio (95% CI)** | **Cut-off** | **Z value** | **P value** |
| --- | --- | --- | --- | --- |
| **Demographics** |  |  |  |  |
| Male sex | 1.177(1.037,1.336) | Present | 2.517 | 0.011842* |
| Age at diagnosis | 1.822(1.815,1.829) | 64.31 | -44.718 | < 2e-16*** |
| **Hospitalization** |  |  |  |  |
| Total number of hospital admissions | 0.9999(0.9992,1.0005) | - | -0.444 | 0.657303 |
| Number of emergency readmissions | 1.0000(0.9918,1.0083) | - | 0.01 | 0.991719 |
| Mean readmission interval (days) | 0.9999(0.9998,1.0001) | - | -0.911 | 0.362353 |
| Cumulative length-of-stay | 1.0007(1.0004,1.0009) | 49 | 5.569 | 2.56E-08*** |
| **Comorbidity** |  |  |  |  |
| Cardiovascular disease | 1.266(1.064,1.507) | Present | 2.664 | 0.000733*** |
| Respiratory disease | 1.093(0.541,2.207) | - | 0.247 | 0.805041 |
| Kidney disease | 1.279(1.125,1.454) | Present | 3.77 | 0.000163*** |
| Endocrine disease | 1.028(0.744,1.421) | - | 0.167 | 0.867187 |
| Diabetes mellitus | 1.208(1.032,1.415) | Present | 2.356 | 0.00018484*** |
| Hypertension | 1.549(0.665,3.608) | Present | 1.014 | 0.0000317*** |
| Gastrointestinal disease | 1.015(0.903,1.141) | - | 0.248 | 0.803777 |
| Obesity | 0.932(0.638,1.361) | - | -0.365 | 0.715469 |
| **Drug prescriptions (mean daily dosage)** |  |  |  |  |
| Cardiac glycosides | 0.940(0.820,1.078) | - | -0.882 | 0.377695 |
| Phosphodiesterase type-3 inhibitors | 1.822(0.818,4.057) | Present | 1.468 | 0.0001449*** |
| Thiazides and related diuretics | 1.024(0.852,1.230) | - | 0.251 | 0.801912 |
| Loop diuretics | 1.380(1.177,1.617) | Present | 3.969 | 7.22E-05*** |
| Potassium-sparing diuretics and aldosterone | 1.063(0.929,1.218) | - | 0.89 | 0.373451 |
| Anti-arrhythmias drugs | 1.042(0.876,1.240) | - | 0.464 | 0.642696 |
| Beta blockers | 0.681(0.598,0.776) | Present | -5.751 | 8.90E-09*** |
| Vasodilator antihypertensive drugs | 1.906(1.609,2.258) | Present | 7.461 | 8.58E-14*** |
| Centrally acting antihypertensive drugs | 1.024(0.759,1.382) | - | 0.158 | 0.874823 |
| Alpha blockers | 0.809(0.668,0.979) | Present | -2.18 | 0.029276* |
| **Laboratory examinations** |  |  |  |  |
| Hemoglobin, g/dL | 1.018(0.892,1.161) | - | 0.266 | 0.789909 |
| Alkaline phosphatase, U/L | 1.001(0.999,1.002) | - | 1.003 | 0.315877 |
| Hematocrit, L/L | 0.040(0.000,13.173) | - | -1.088 | 0.276436 |
| Lymphocyte, x10^9/L | 0.812(0.748,0.882) | 2.11 | -4.927 | 8.34E-07*** |
| Neutrophil, x10^9/L | 1.063(1.049,1.077) | 2.78 | 9.156 | < 2e-16*** |
| Platelet, x10^9/L | 0.999(0.998,1.000) | 296 | -2.512 | 0.012014* |
| APTT, secs | 1.005(0.999,1.010) | - | 1.739 | 0.08209. |
| INR | 0.953(0.662,1.372) | - | -0.259 | 0.795395 |
| Prothrombin time, sec | 1.029(1.000,1.059) | - | 1.944 | 0.051924. |
| Red cell count, x10^12/L | 1.409(1.078,1.8418) | 3.64 | 2.513 | 0.00011971*** |
| Total protein, g/L | 0.998(0.992,1.004) | - | -0.667 | 0.504628 |
| Total bilirubin, umol/L | 1.002(1.001,1.004) | 47 | 3.264 | 0.001098** |
| Red cell distance width, % | 1.069(1.045,1.094) | 14.5 | 5.745 | 9.20E-09*** |
| Mean cell volume, fL | 1.040(1.021,1.060) | 43.1 | 4.061 | 4.88E-05*** |
| Mean cell hemoglobin concentration, g/dL | 0.921(0.875,0.969) | 34.5 | -3.173 | 0.001508** |

**Supplementary Table 5B. Electronic frailty index for all-cause mortality risk prediction derived from the multivariable Cox regression analysis.**

PHTN: pulmonary hypertension; LOS: Length of stay.

| **Characteristics** | **Cut-off** | **Point** |
| --- | --- | --- |
| Male sex | Present | 1 |
| Age at diagnosis | >64.3 | 1 |
| Cumulative length-of-stay | >49 | 1 |
| Cardiovascular disease | Present | 1 |
| Kidney disease | Present | 1 |
| Diabetes mellitus | Present | 1 |
| Hypertension | Present | 1.5 |
| Lymphocyte, x10^9/L | <2.11 | 1 |
| Neutrophil, x10^9/L | >2.78 | 1 |
| Platelet, x10^9/L | <296 | 1 |
| Red cell count, x10^12/L | >3.64 | 1 |
| Total bilirubin, umol/L | >47 | 1 |
| Red cell distance width, % | >14.5 | 1 |
| Mean cell volume, fL | >43.1 | 1 |
| Mean cell hemoglobin concentration, g/dL | <34.5 | 1 |

**Supplementary Figure 1. Kaplan-Meier curves for cardiovascular, renal and diabetic complications.**


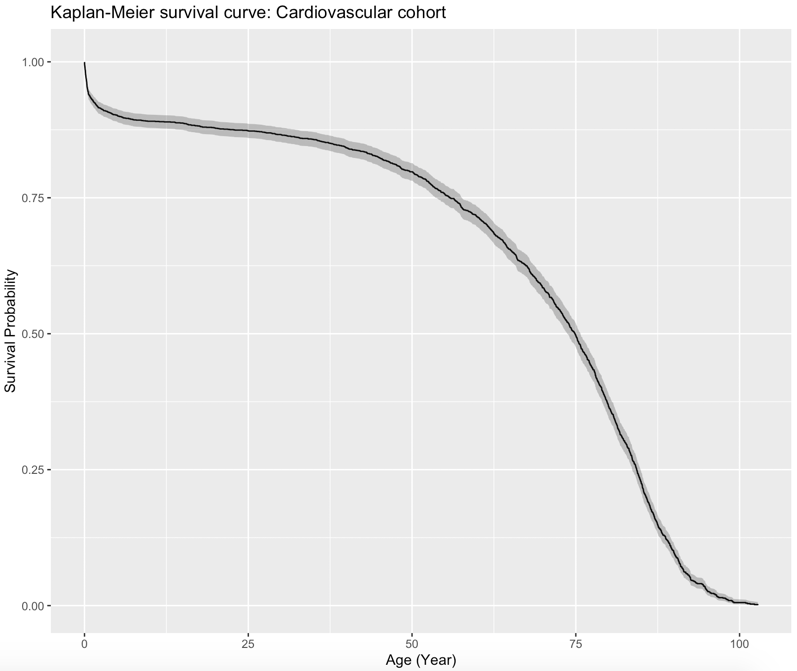

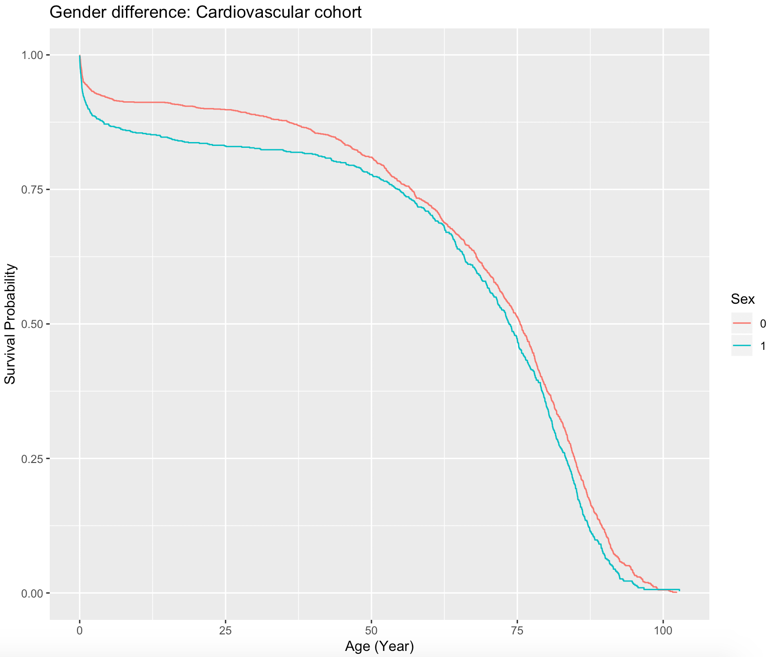


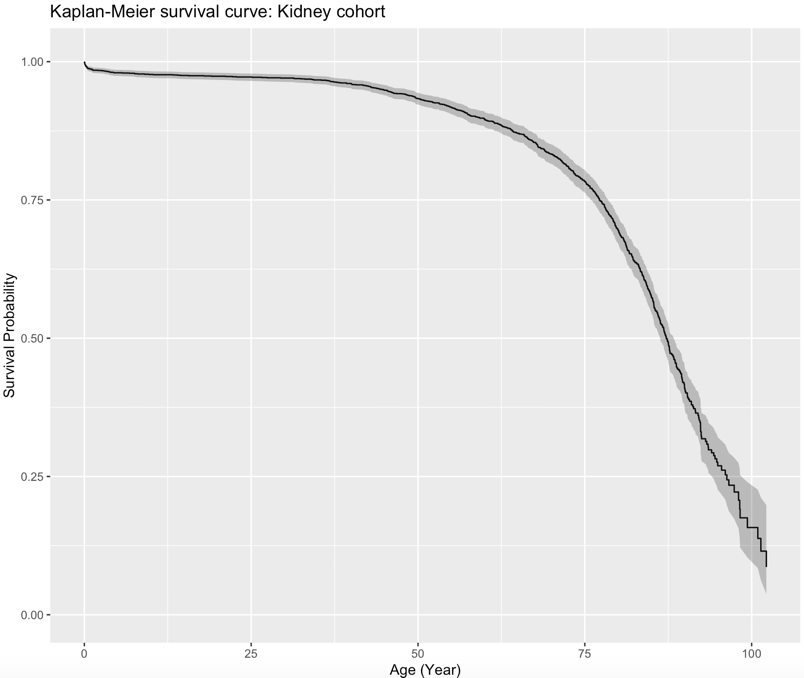

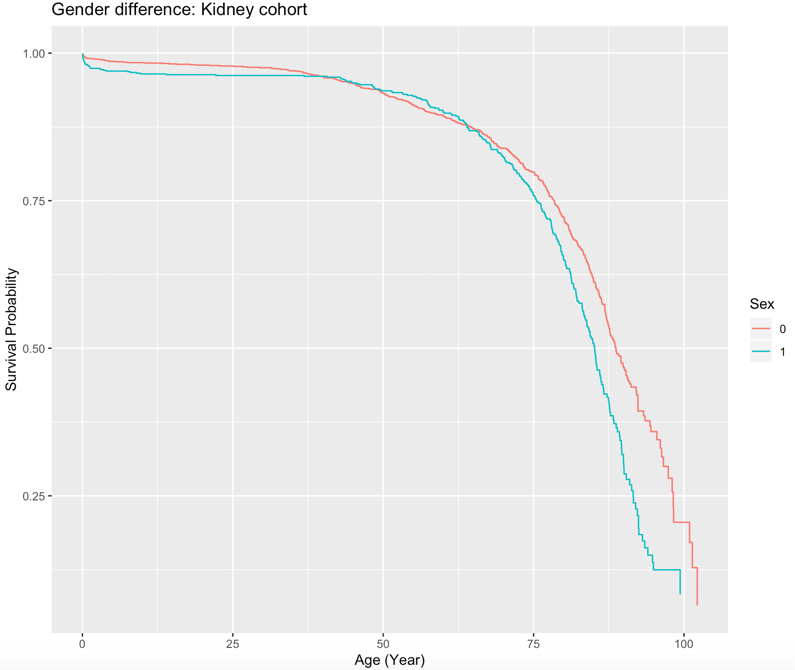


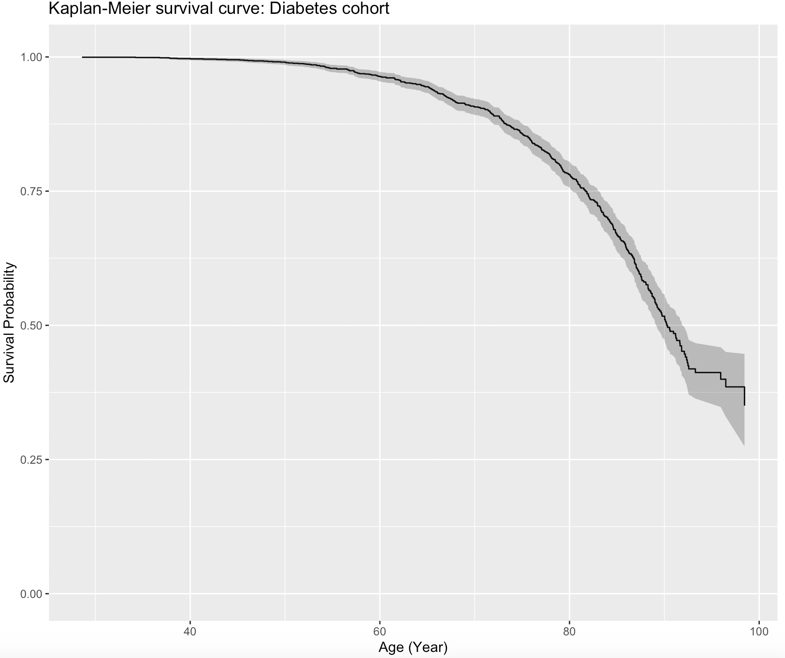

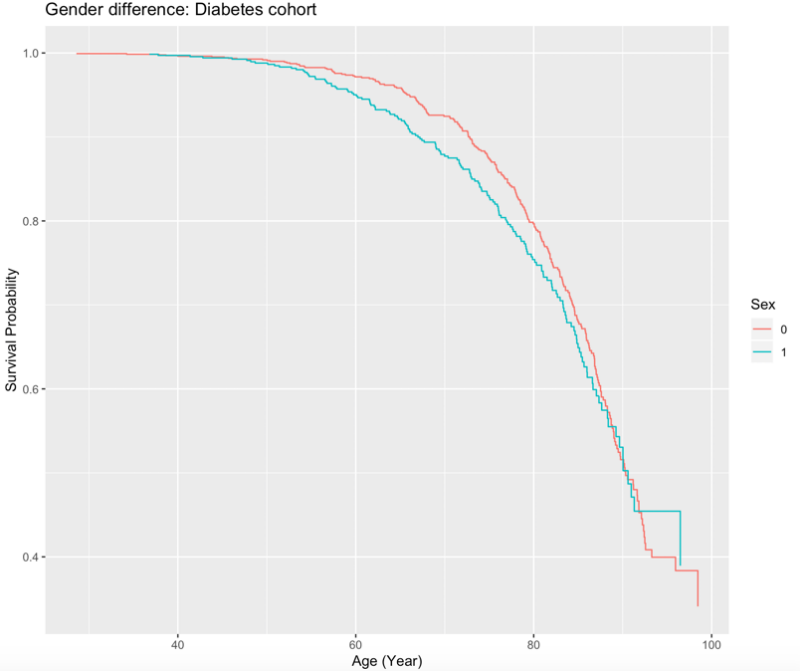


**Supplementary Figure 2. The hazard ratios of the variables predicting all-cause mortality derived from the multivariable Cox model**

APTT: Activated partial thromboplastin time; INR: International normalized ratio; IQR: Interquartile range; LOS: Length of stay; MCV: Mean corpuscular volume; MCH: Mean corpuscular hemoglobin; PHTN: Pulmonary hypertension.


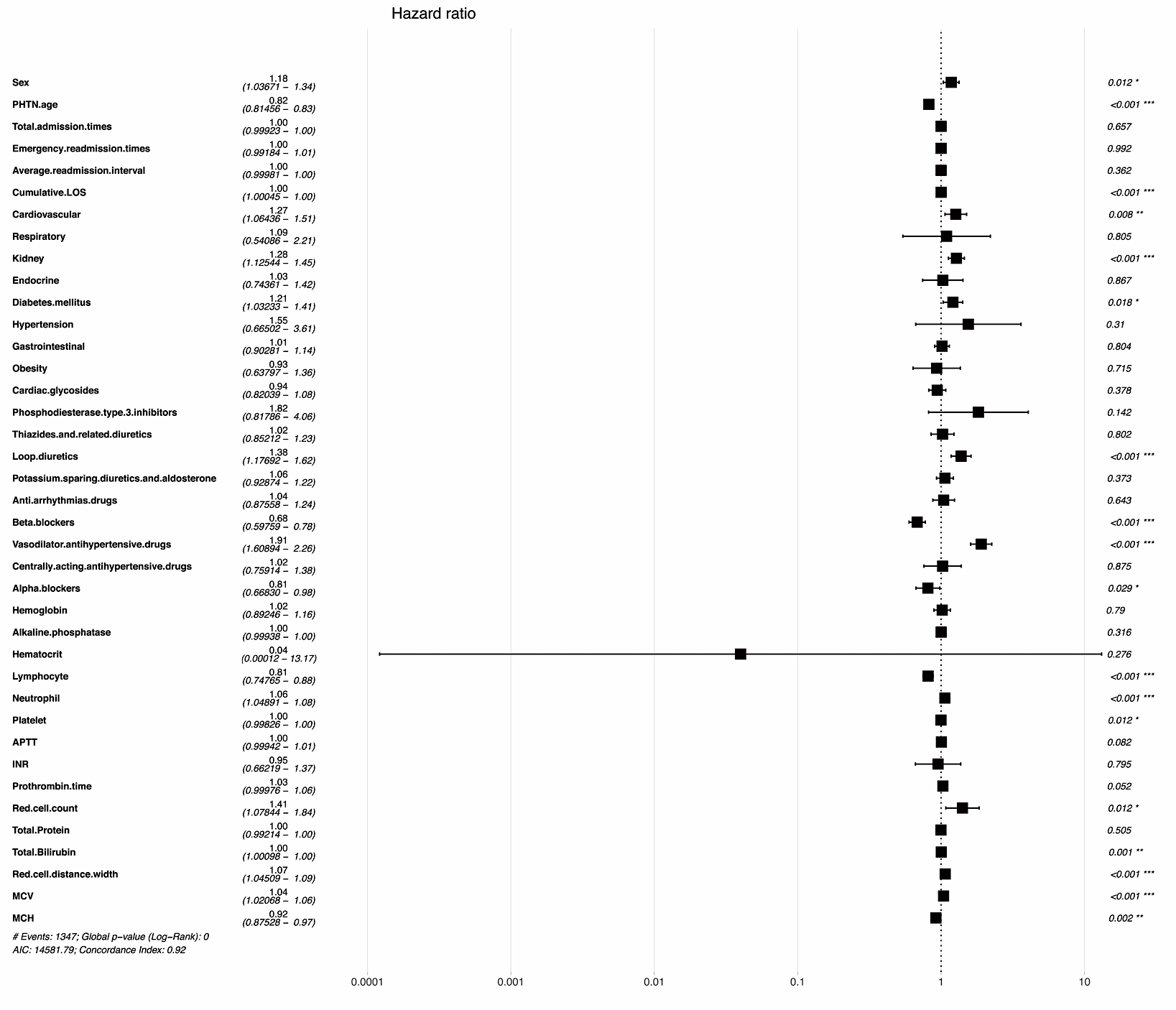


**Supplementary Figure 3. The hazard ratios of the variables predicting complications of cardiovascular, kidney, and diabetes derived from the multivariable Cox model**

APTT: Activated partial thromboplastin time; INR: International normalized ratio; IQR: Interquartile range; LOS: Length of stay; PHTN: Pulmonary hypertension.


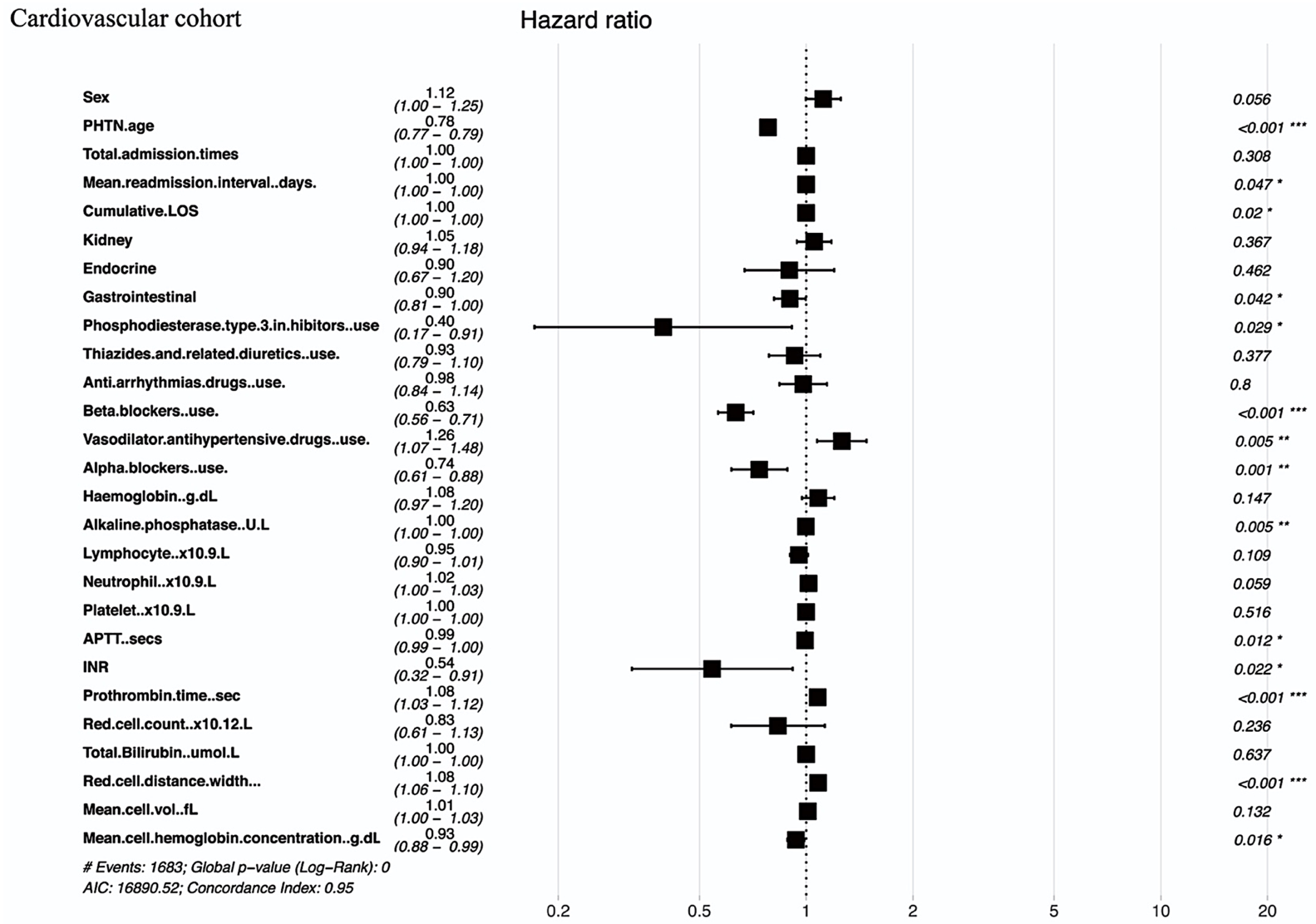


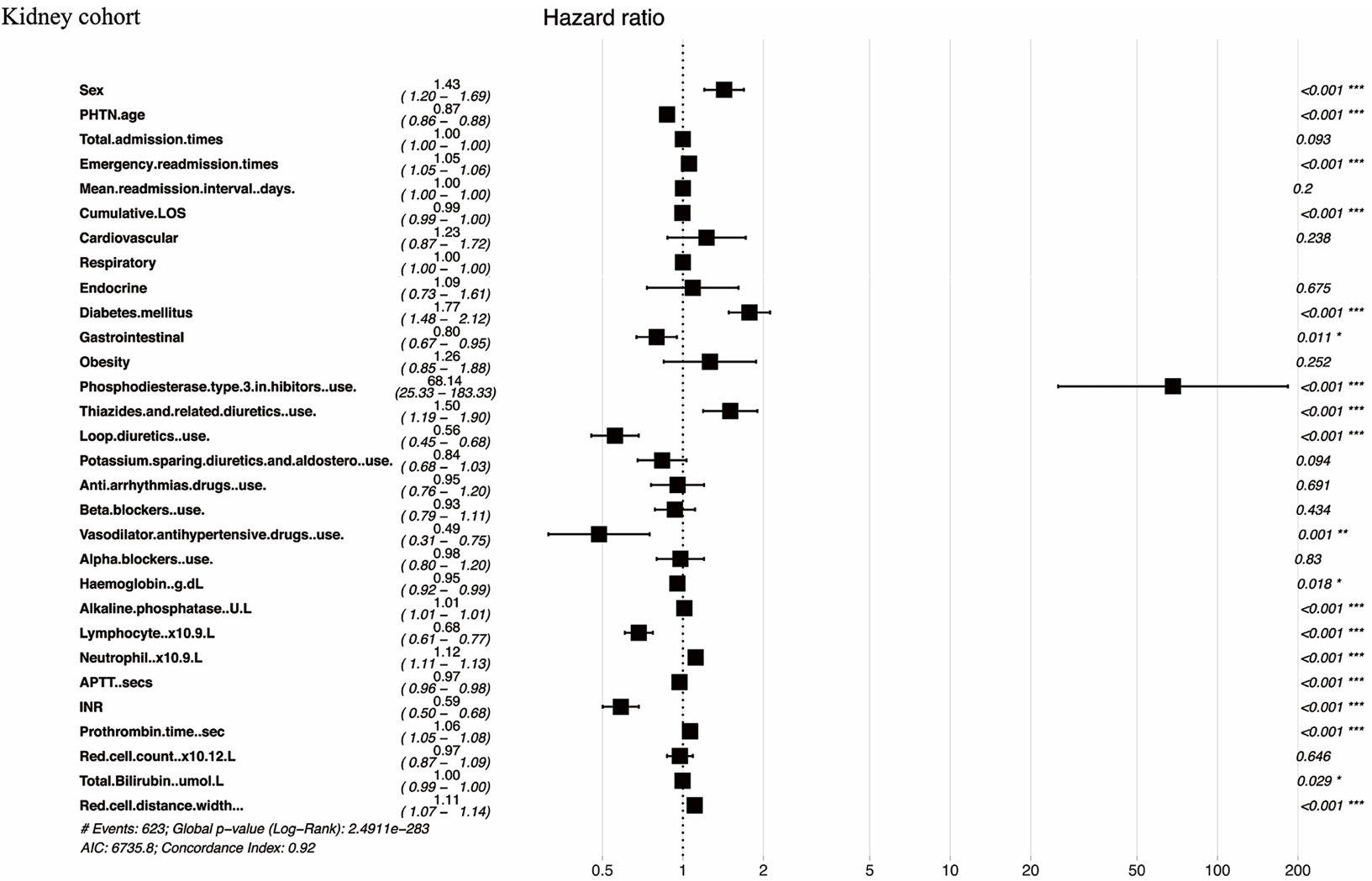


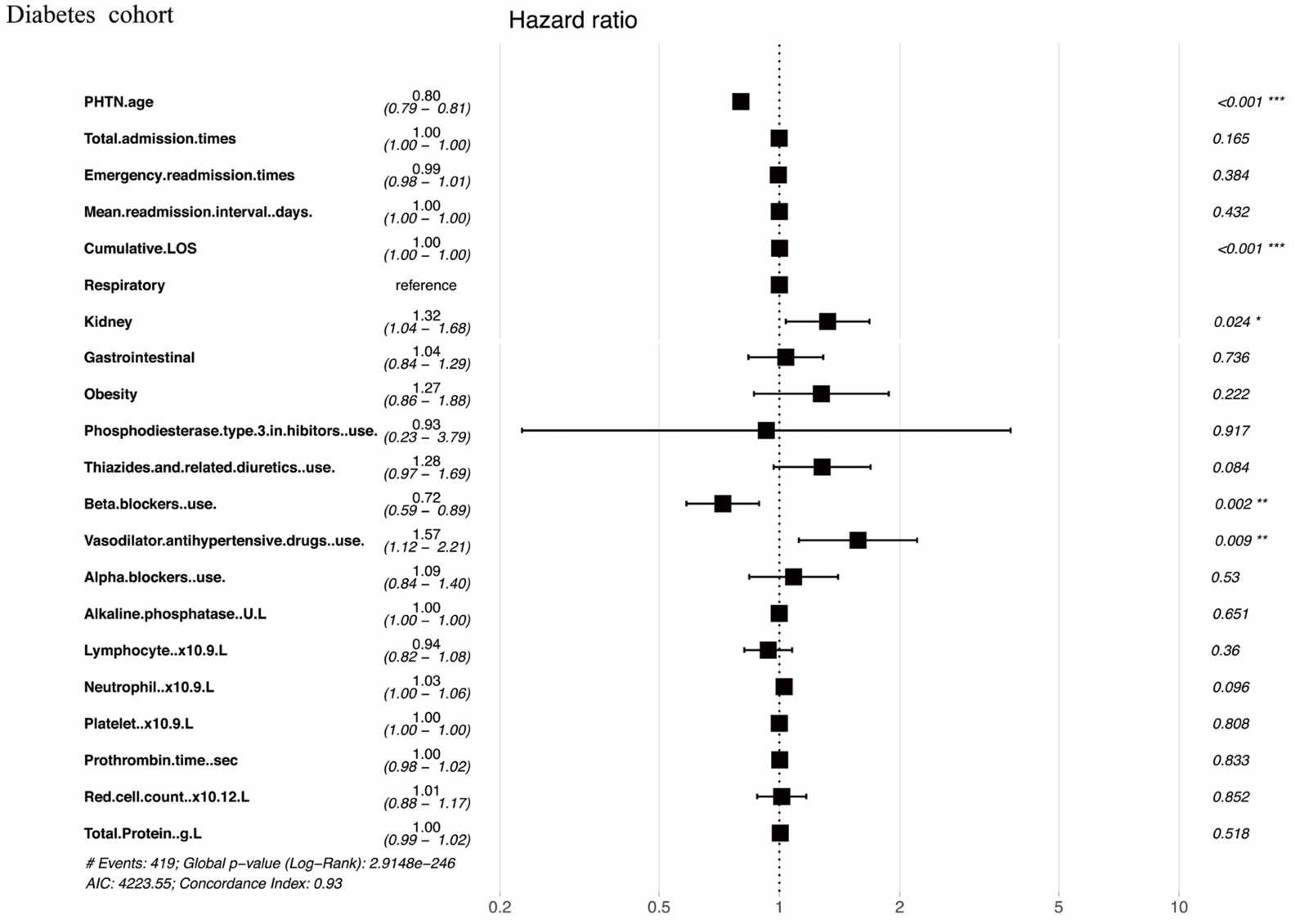


**Supplementary Figure 4. Interaction importance of demographics characteristics with other variables to predict all-cause mortality**

**Supplementary Figure 5. Interaction importance of comorbidities with other variables to predict all-cause mortality**

**Supplementary Figure 6. Interaction importance of drug prescription characteristics with other variables to predict all-cause mortality**

**Supplementary Figure 7. Interaction importance of laboratory examinations with other variables to predict all-cause mortality**

**Supplementary Figure 8. Minimal depth ranking of variables with random survival forest model to predict all-cause mortality**

APTT: Activated partial thromboplastin time; INR: International normalized ratio; IQR: Interquartile range; LOS: Length of stay; MCV: Mean corpuscular volume; MCH: Mean corpuscular hemoglobin; PHTN: Pulmonary hypertension.

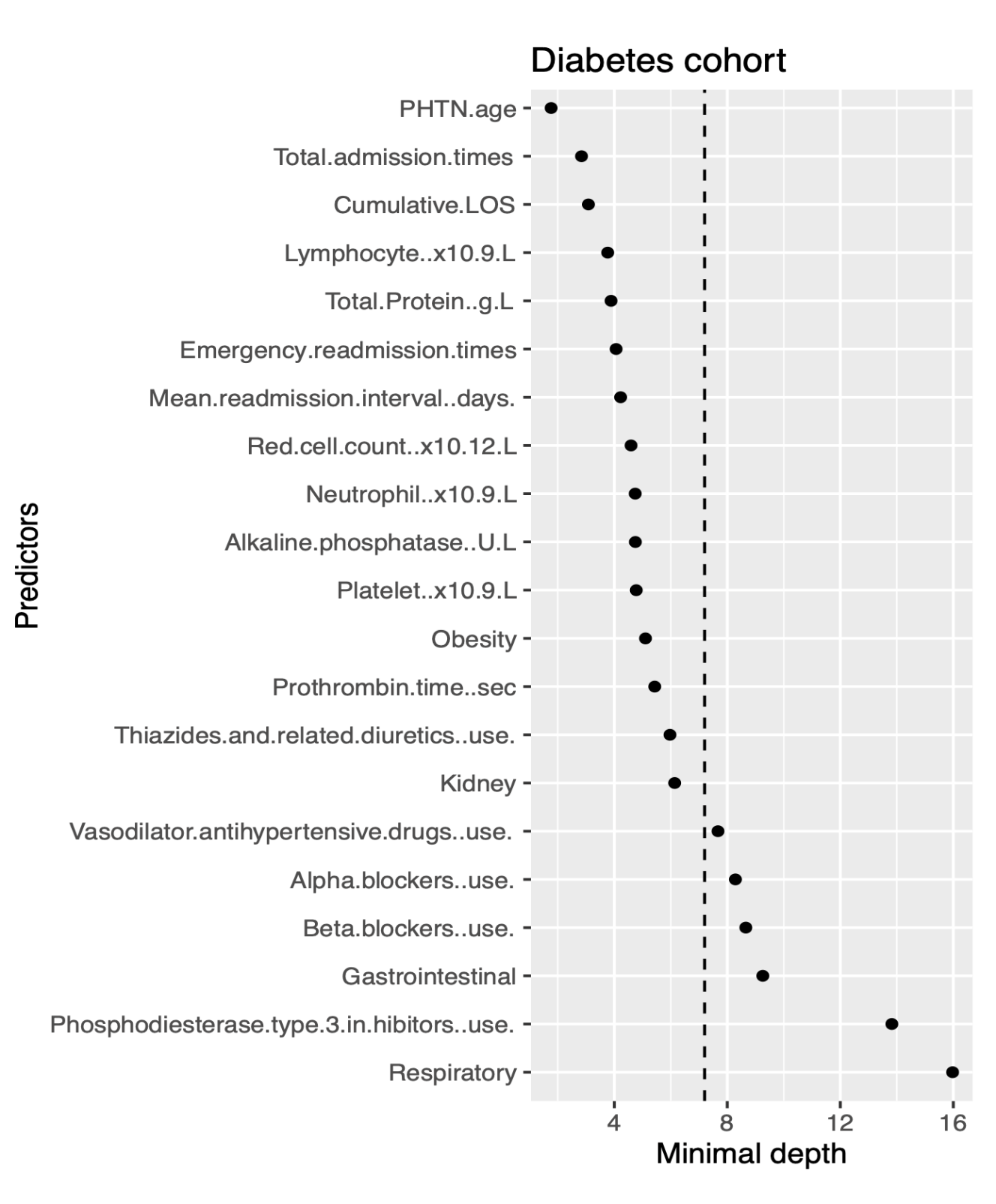

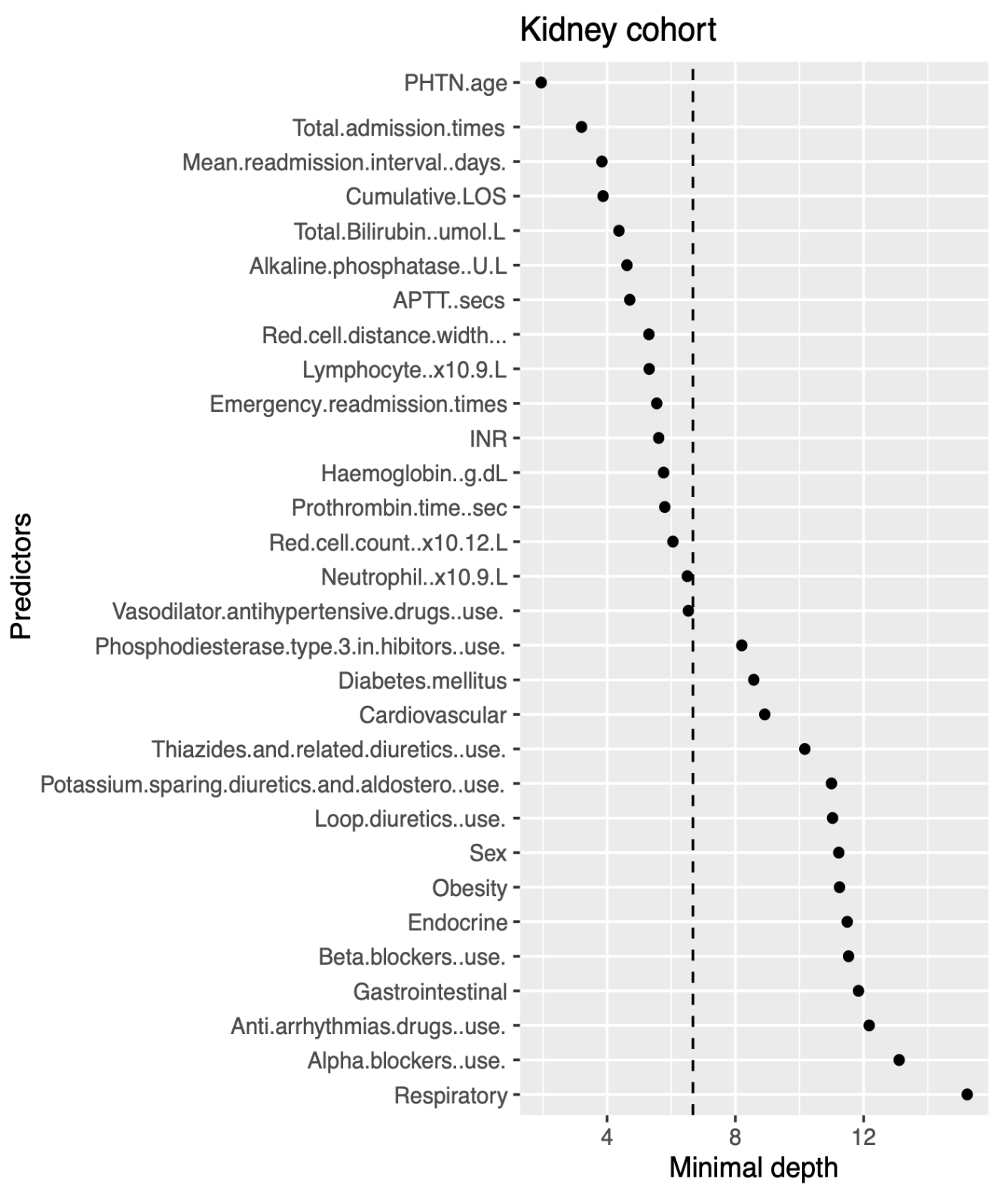

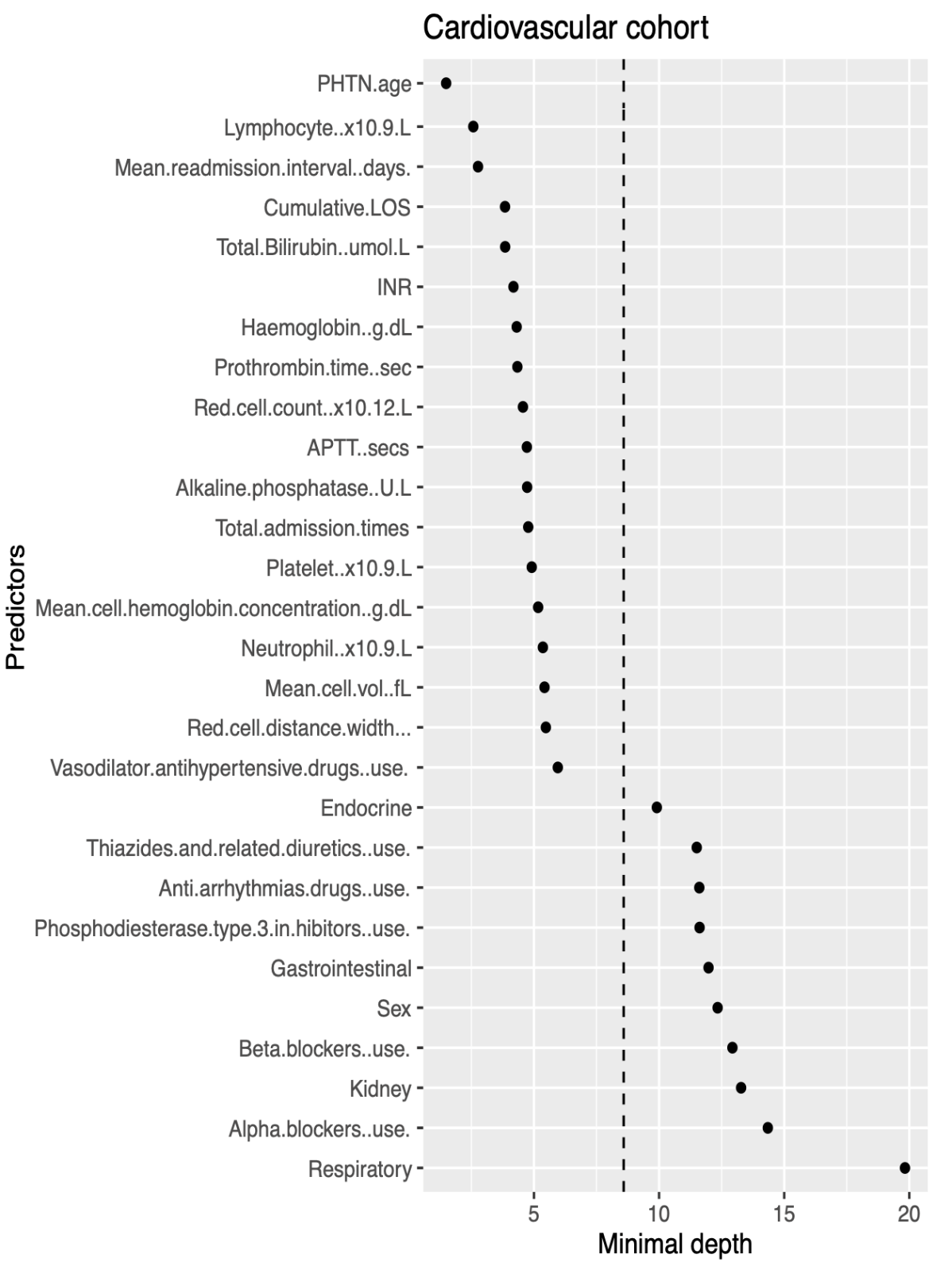


**Supplementary Figure 9. Histogram of electronic frailty index stratified by mortality risk of patients with PHTN.**

**Supplementary Figure 10. Marginal effects of the constructed electronic frality index to predict all-cause mortality**
